# Executioner caspases 3 and 7 are dispensable for intestinal epithelium turnover and homeostasis at steady state

**DOI:** 10.1073/pnas.2024508119

**Published:** 2022-02-01

**Authors:** Farzaneh Ghazavi, Jelle Huysentruyt, Jordy De Coninck, Stephanie Kourula, Sofie Martens, Behrouz Hassannia, Tim Wartewig, Tatyana Divert, Ria Roelandt, Bastian Popper, Andreas Hiergeist, Peter Tougaard, Tom Vanden Berghe, Marie Joossens, Geert Berx, Nozomi Takahashi, Adam Wahida, Peter Vandenabeele

**Affiliations:** ^a^VIB-UGent Center for Inflammation Research, B-9052 Ghent, Belgium;; ^b^Department of Biomedical Molecular Biology, Ghent University, B-9052 Ghent, Belgium;; ^c^Molecular and Cellular Oncology Laboratory, Department of Biomedical Molecular Biology, Ghent University, B-9052 Ghent, Belgium;; ^d^Cancer Research Institute Ghent, B-9052 Ghent, Belgium;; ^e^Center of Molecular and Cellular Oncology, Yale Cancer Center, Yale School of Medicine, New Haven, CT 06511;; ^f^Core Facility Animal Models, Biomedical Center, Ludwig Maximilians University of Munich, D-82152 Planegg-Martinsried, Germany;; ^g^Institute of Pathology, Technical University of Munich, D-81675 Munich, Germany;; ^h^Institute of Clinical Microbiology and Hygiene, University Hospital Regensburg, D-93053 Regensburg, Germany;; ^i^Laboratory of Pathophysiology, Faculty of Biomedical Sciences, University of Antwerp, B-2610 Wilrijk, Belgium;; ^j^Laboratory of Microbiology, Department of Biochemistry and Microbiology, Faculty of Science, Ghent University, B-9000 Ghent, Belgium;; ^k^Department of Medicine III, Klinikum rechts der Isar, TUM School of Medicine, Technical University of Munich, D-81675 Munich, Germany;; ^l^Institute of Metabolism and Cell Death, Helmholtz Zentrum München, D-85764 Neuherberg, Germany

**Keywords:** mucosal immunology, apoptosis, caspases, cell death, regeneration

## Abstract

Apoptosis is widely believed to be crucial for epithelial cell death and shedding in the intestine, thereby shaping the overall architecture of the gastrointestinal tract, but also regulating tolerance induction, pinpointing a role of apoptosis intestinal epithelial cell (IEC) turnover and maintenance of barrier function, and in maintaining immune homeostasis. To experimentally address this concept, we generated IEC-specific knockout mice that lack both executioner caspase-3 and caspase-7 (*Casp3/7*^ΔIEC^), which are the converging point of the extrinsic and intrinsic apoptotic pathway. Surprisingly, the overall architecture, cellular landscape, and proliferation rate remained unchanged in these mice. However, nonapoptotic cell extrusion was increased in *Casp3/7*^ΔIEC^ mice, compensating apoptosis deficiency, maintaining the same physiological level of IEC shedding. Microbiome richness and composition stayed unaffected, bearing no sign of dysbiosis. Transcriptome and single-cell RNA sequencing analyses of IECs and immune cells revealed no differences in signaling pathways of differentiation and inflammation. These findings demonstrate that during homeostasis, apoptosis per se is dispensable for IEC turnover at the top of intestinal villi intestinal tissue dynamics, microbiome, and immune cell composition.

## Significance

Historically, programmed cell death by apoptosis is considered crucial for proper intestinal organogenesis and gut homeostasis. To challenge this concept, we generated caspase-3 and -7 double knockout mice specifically in intestinal epithelial cells (IECs). However, absence of apoptosis in IECs elicits neither morphological and inflammatory changes nor intestinal dysbiosis during gut homeostasis at steady state. This demonstrates the robustness of intestinal homeostasis at steady state for the absence of caspase-3/7 and shows that in contrast to caspase-8, which keeps necroptosis and associated inflammation in check, caspase-3/7–dependent apoptosis of IECs in homeostatic conditions is dispensable for normal intestinal development, immune cell composition, and microbiome control.

Tissue homeostasis requires a fine-tuned balance between self-renewal, proliferation, differentiation, and ultimately cell death (1). A discrete single epithelial cell layer in the gut forms the barrier between luminal content and the intestinal mucosa and functions in digestion and absorption. These epithelial cells all arise from actively cycling LGR5^+^ stem cells at the bottom of intestinal crypts, subsequently moving up the crypt–villus axis and differentiating into specialized absorptive, secretory, or defensive epithelial lineages (2, 3). Constant cell division enables rapid turnover and apical-oriented migration of these cells, resulting in a turnover time of 3 to 5 d. Under homeostatic conditions, cellular turnover in the intestinal epithelium—through the shedding of cells at the apical villi—accounts for approximately 10% of the dying cells in our body. Apoptosis is widely believed to be the critical modality for epithelial cell death and subsequent shedding, thus shaping the overall architecture of the gastrointestinal tract and regulating tolerance induction toward food and microbiome during homeostasis at steady state (4, 5).

Biologically, apoptosis is viewed as immunologically silent, serving as a prerequisite for eliminating old or unneeded cells without causing damage to the surrounding tissues by initiating aberrant immune responses. Moreover, apoptotic cells serve as a critical source of signals required for wound healing and regeneration in multiple organisms (6–8). The concept of apoptosis-induced proliferation (AiP) following injury (9–12) exemplifies its essential homeostatic functions via the release of attractive factors for stem and immune cells (13–15). Mechanistic studies recently evoked that specific metabolites released from apoptotic dying cells function as tissue messengers (16). Seminal work from Blander and coworkers documented sampling of the preapoptotic cell content prior to cell extrusion at the top of the villi by the CD103^+^ subset of macrophages and dendritic cells, which form protrusions toward the shedding cells (17). This recognition resulted in antiinflammatory signaling in the innate immune cells of the submucosa layer. This phenomenon suggests that apoptotic cell shedding might represent inward cross-talk to the mucosal immune system, which might be critical for tolerance induction. On the other hand, the daily stream of dying cells shed into the lumen may denote outward cross-talk to the gut microbiome by affecting the composition of microbial communities, a principle that recently was shown in cell death–microbiome interaction in *Drosophila* (18).

Alternatively, disturbances within this highly regulated dynamic system, where cell proliferation must be counterbalanced by cell death, can cause serious diseases. Resistance to cell death is believed to be a driving force of intestinal tumor development (19). On the contrary, excessive cell death in the gut might eventually result in barrier disruption and chronic inflammation (20). Altogether, these findings support an emerging concept in which aberrant cell death and survival signaling pathways in the gut constitute an overarching feature of intestinal disease such as inflammatory bowel disease, irrespective of the genetic background (21–24). Indeed, monogenetic variants in members of the TNF- or NF-κB–signaling pathway such as in RIPK1, NEMO, and caspase-8, which impair proper downstream signaling and skew TNF signaling toward excessive cell death and intestinal inflammation (25–28). Most prominently, conditionally deleting caspase-8 in intestinal epithelial cells (IECs) led to terminal ileitis due to excessive necroptosis (29). Caspase-8 has a dual function in cell death regulation, serving as an initiator caspase in both intrinsic and extrinsic apoptosis, and acting as a dominant-negative regulator of TNF-induced necroptosis by cleaving RIPK1 and avoiding excessive necroptosis induction (30–32). In contrast to caspase-8, inhibition or deletion of caspase-3 or caspase-7 does not sensitize toward TNF-induced necroptosis (33, 34).

Intriguingly, while homeostatic apoptosis of epithelial cells is considered essential for IEC turnover, where it counterbalances cell proliferation in the intestinal crypts and subsequently determines the number and differentiation of epithelial cells shaping the GI tract architecture (15, 35), Brinkman et al. showed that intestinal morphology of mice deficient for either caspase-3 or caspase-7 did not differ from wild-type (WT) mice (36). However, single deletion of one of these executioner caspases might not be sufficient to entirely impair apoptosis, due to their functional redundancy during apoptosis (37). Therefore, it is still unclear whether the apoptotic cell death process as such is essential for morphogenesis during intestinal development. Furthermore, homeostatic cell shedding might occur at least functionally independently of apoptotic cell death initiation by overcrowding of epithelial cells at the top of the villi. This in turn induces the extrusion of living cells through mechanical force exerted by neighboring epithelial cells likely followed by anoikis once the interaction with neighboring cells is lost (38, 39). However, it remains contentious whether cell death precedes the dynamic extrusion process or whether cell death occurs at a later time point, before the release of epithelial cells into the lumen (38–41).

To investigate the role of apoptosis in intestinal homeostasis, we generated tissue-specific knockout mice that lack both caspase-3 and caspase-7 in the intestinal epithelium (*Casp3/7*^ΔIEC^). Overall, we found that absence of executioner caspases does not affect intestinal architecture, immune cell composition, and microbiome composition, illustrating genetically the robustness of intestinal homeostasis at steady state to bypass the requirement of such a complex and regulated process as caspase-3 and -7–driven apoptosis (35, 38, 40, 42, 43).

## Results

### Epithelial Deletion of Caspase-3 and -7 Does Not Affect Intestinal Homeostasis at Steady State.

**Fig. 1.**
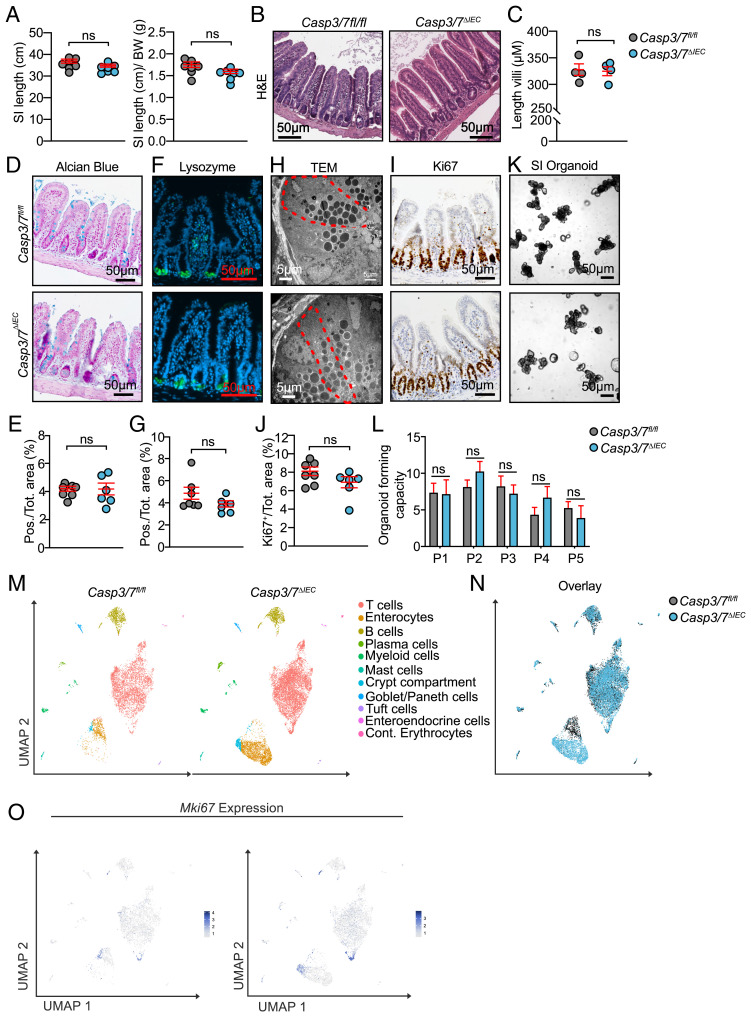
Epithelial deletion of Caspase-3 and -7 does not affect intestinal homeostasis at steady state. (*A*) The length of small intestine and its normalized value to the body weight in *Casp3/7*^ΔIEC^ (*n* = 7) were compared to *Casp3/7*^fl/fl^ mice (*n* = 7). (*B*) Representative micrographs of H&E staining of small intestinal sections from *Casp3/7*^ΔIEC^ and *Casp3/7*^fl/fl^ mice. Scale bars are indicated. (*C*) Quantification of the average length of around 200 villi measured in *Casp3/7*^ΔIEC^ (*n* = 4) and *Casp3/7*^fl/fl^ (*n* = 4) mice. (*D* and *E*) Alcian Blue staining (as a proxy for goblet cells) and automated quantification thereof in small intestinal sections from *Casp3/7*^ΔIEC^ (*n* = 6) and *Casp3/7*^fl/fl^ (*n* = 7) mice. (*F* and *G*) Lysozyme staining (as a proxy for Paneth cells) and automated quantification thereof in small intestinal sections from *Casp3/7*^ΔIEC^ (*n* = 6) and *Casp3/7*^fl/fl^ (*n* = 7). (*H*) Ultramicroscopic analysis of Paneth cell compartment via TEM. (*I* and *J*) Ki67 staining (as a proxy for proliferating cells) and automated quantification thereof in small intestinal sections from *Casp3/7*^ΔIEC^ (*n* = 6) and *Casp3/7*^fl/fl^ (*n* = 7) mice. (*K* and *L*) The organoid cultures derived from LGR5^+^ intestinal stem cells of *Casp3/7*^ΔIEC^ and *Casp3/7*^fl/fl^ mice and organoid forming capacity calculated after the first five passages (P1 to P5). (Scale bars, 50 µm.) A two-tailed unpaired *t* test was conducted to compare *Casp3/7*^ΔIEC^ with *Casp3/7*^fl/fl^ mice. Each point represents an individual mouse, and the line represents mean ± SEM. For bar plots in *L*, the error bars are the SEM and three mice per group per passage were analyzed. (*M*) UMAP of intraepithelial (IEL) cells extracted from three *Casp3/7*^fl/fl^ and *Casp3/7*^ΔIEC^ (*n* = 3 per genotype) mice. Identified populations are indicated. In total, 679 *Casp3/7*^fl/fl^ vs. 2,227 *Casp3/7*^ΔIEC^ enterocytes; when looking at total cells per sample: 7,584 *Casp3/7*^fl/fl^ cells and 11,956 *Casp3/7*^ΔIEC^ cells, equaling a relative share of 9.0% in WT and 18.6% in *Casp3/7*^ΔIEC^. The relative higher share of enterocytes in the case of Casp3/7^ΔIEC^ cells is probably due to the stressful conditions during the isolation procedure resulting in higher levels of apoptosis in WT cells, while this occurs less in enterocytes derived from *Casp3/7*^ΔIEC^ mice. (*N*) Overlay of *Casp3/7*^fl/fl^ and *Casp3/7*^ΔIEC^ cells from IEL cells. (*O*) UMAP with normalized expression of *Mki67*. (ns: non significant *P-*value.)

To evaluate the role of apoptosis in IEC homeostasis at steady state, we generated mice deficient for the executioners of apoptotic cell death, caspase-3 and -7 (*Casp3/7*^ΔIEC^) in the intestinal epithelium, using the expression of the Cre-recombinase under the control of an IEC-specific promoter (Villin-Cre) (*SI Appendix*, Fig. S1 *A* and *B*). These mice were viable, born at the expected Mendelian ratios, and reached adulthood. When examined for the overall gut architecture and histopathology, the *Casp3/7*^ΔIEC^ mice showed no significant differences in the length of the small intestine and colon or in their normalized value to the bodyweight between *Casp3/7*^ΔIEC^ and littermate *Casp3/7*^fl/fl^ (henceforth denoted as WT) mice (Fig. 1*A*). Despite the widely accepted paradigm that apoptosis is critical for intestinal cellular turnover (15, 35), histomorphological analysis by hematoxylin and eosin (H&E) staining revealed that loss of both caspase-3 and -7 had no apparent effect on the overall intestinal architecture (Fig. 1*B*). Specifically, morphometry of villi and crypts showed no shortening or blunting of small intestinal villi (Fig. 1*C*). We next examined via histological analysis IEC differentiation, a fundamental cornerstone of homeostatic cell turnover (Fig. 1 *D*, *F*, and *I*). Analysis of goblet and Paneth cells at adult age or during development by staining mucin and lysozyme, respectively, revealed no changes in goblet or Paneth cell abundances in *Casp3/7*^ΔIEC^ mice (Fig. 1 *E* and *G* and *SI Appendix*, Fig. S1*C*). To examine intracellular morphological changes or subcellular signs of cellular stress due to the absence of apoptosis in the IEC compartment, we performed transmission electron microscopic (TEM) analysis of small intestinal crypts and the Paneth cell compartment (Fig. 1*H*). Again, no overt signs of Paneth or goblet cell impairment in *Casp3/7*^ΔIEC^ mice were seen. Next, we examined the proliferative capacity of IECs in *Casp3/7*^ΔIEC^ and WT mice. By staining and quantifying Ki67^+^ cells in small intestinal sections, no significant difference was observed (Fig. 1 *I* and *J*). To functionally address the intrinsic cell differentiation and regeneration potential of stem cells, we employed the reductionist small intestinal organoid system starting from isolated crypts harboring LGR5^+^ intestinal stem cells (44). Organoids derived from either adult stem cells of *Casp3/7*^ΔIEC^ or WT mice showed the same preserved crypt–villus architecture with crypt-like budding domains, which led to a similar organoid-forming capacity between *Casp3/7*^ΔIEC^ and WT cultures (Fig. 1 *K* and *L*). Altogether, these data imply that the net result of the inter- and intracellular processes that determine growth and differentiation of intestinal epithelial cells revealed no phenotypic differences between WT and *Casp3/7*^ΔIEC^ mice.

To further challenge this overall conclusion, we performed single-cell RNA sequencing (scRNA-seq) on cells isolated from the small intestine, colon, and mesenteric lymph nodes. In the small intestine, we analyzed cells originating from the superficial mucosal layer comprising mostly IECs and from the lamina propria, mainly containing intraepithelial leukocytes. Quantitative analysis portraying the cellular diversity and size of individual populations revealed no marked difference between *Casp3/7*^ΔIEC^ and WT mice (Fig. 1*M*). Overlay depiction of Uniform Manifold Approximation and Projections (UMAPs) obtained after annotation using cell type–specific expression markers indicated that almost all cell types identified via scRNA-seq harbored a near-perfect overlap (Fig. 1*N*). When considering epithelial populations, we noted that compared to WT mice, the samples obtained from *Casp3/7*^ΔIEC^ contained significantly more cells (Fig. 1*N*). Differential gene expression analysis revealed that the difference between both genotypes mainly included transcripts of mitochondrial genes or genes involved in oxidative phosphorylation. This effect is likely due to the observed differential surviving cells between WT and epithelial cells because of the extensive digestion protocol during isolation, encompassing lower levels of cellular demise of *Casp3/7*^ΔIEC^ intestinal cells, which are unable to undergo apoptosis during the isolation procedure. This mitochondrial signature may have resulted from differential stress adaptive mitochondrial oxidative phosphorylation system (OXPHOS) responses in apoptosis-deficient surviving cells. We hypothesize that cells with an apoptosis-proficient genotype would be eliminated, while those with an apoptosis-deficient genotype instead accumulate stress response signatures. But as said above, neither the number of differentiated cells nor their characteristic gene expression pattern showed any difference between WT and *Casp3/7*^ΔIEC^ IECs.

Finally, we confirmed the immunohistochemical analysis of the proliferative compartment in gut via Ki67^+^ cells, by assessing no difference in *Mki67* expression in all cell types isolated from the superficial mucosal layers from both genotypes (Fig. 1*O*).

Next, we analyzed the colon. Macroscopic or histomorphological analysis of the colon of *Casp3/7*^ΔIEC^ mice revealed no differences compared to WT (*SI Appendix*, Fig. S2 *A* and *B*). Moreover, absolute and relative lengths of the colon were unaltered (*SI Appendix*, Fig. S2 *C* and *D*). Moreover, colonoscopic analysis in aged *Casp3/7*^ΔIEC^ mice revealed no apparent signs of inflammation, neoplastic tissue, or other aberrant tissue architecture, arguing against overt inflammatory or dysplastic processes in the absence of caspase-3 and -7 even at an older age (64 wk) (*SI Appendix*, Fig. S2*E*). Finally, we also examined the cellular landscape in the colon by scRNA-seq analysis. Similar to our analysis in the small intestine, we observed neither qualitative nor quantitative differences in colonic cell composition between WT and *Casp3/7*^ΔIEC^ mice (*SI Appendix*, Fig. S2*F*). Altogether these data suggest that deficiency of major executioner caspases in IECs per se does not affect overall histology of small intestine and colon.

### Apical Shedding of IECs Still Proceeds Independently of Caspase-3/7.

**Fig. 2.**
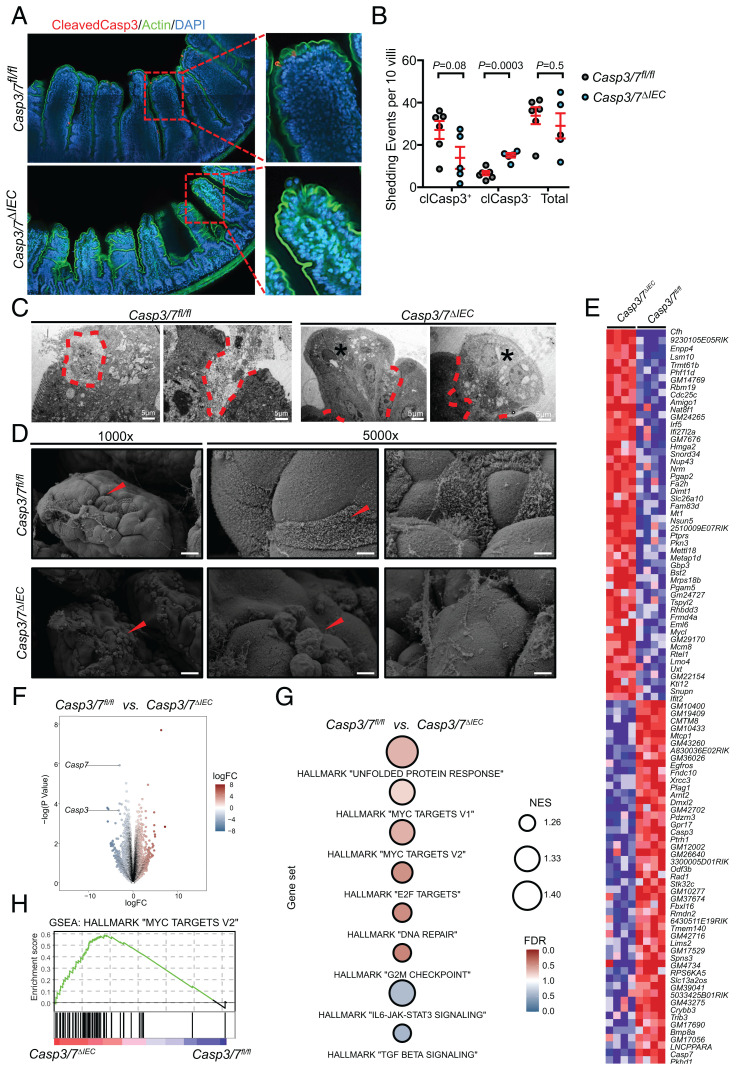
Apical shedding of IECs proceeds independently of Caspase-3 and -7. (*A* and *B*) Nonapoptotic (cleaved caspase-3^−^) and apoptotic (cleaved caspase-3^+^) extrusions were detected by confocal microscopy and quantified in *Casp3/7*^ΔIEC^ (*n* = 5) and *Casp3/7*^fl/fl^ (*n* = 6) mice. Quantification of shedding events is representative of two independent experiments. (*C* and *D*) Ultrastructural analysis of extruding cells at apical villi using TEM and SEM. Scale bars are shown. Asterisks and demarcation show the live and apoptotic extruding cells, respectively. A two-tailed unpaired *t* test was conducted to compare *Casp3/7*^ΔIEC^ with *Casp3/7*^fl/fl^ mice. Each point represents an individual mouse, and the line represents mean ± SEM. (*E*) Top 50 differentially expressed genes between *Casp3/7*^ΔIEC^ and *Casp3/7*^fl/fl^ cells (*n* = 4 biological replicates per group). Expression values are z-score normalized. The gene symbols are indicated on the *Right*. (*F*) Global differential gene expression analysis of RNA-seq data derived from *Casp3/7*^ΔIEC^ and *Casp3/7*^fl/fl^ cells (*n* = 4 biological replicates per group). Data points for *Casp3* and *Casp7* are labeled. LogFC, log2(fold change). (*G*) Gene set enrichment analyses (GSEA) for the indicated signatures. False discovery rate (FDR), color intensity of the circle. Normalized enrichment score (NES), circle diameter. Blue/red indicates the group in which a signature was positively enriched. (*H*) GSEA enrichment plot for the Hallmark gene set “Myc Targets v2.” The genotype is indicated in blue/red.

The above observations reveal that executioner caspases are dispensable in shaping intestinal architecture under steady-state conditions. The question is whether cellular turnover in the intestinal epithelium proceeds independently of IEC apoptosis. Cell death in intestinal homeostasis has been primarily viewed through the prism of cellular shedding during turnover (23). The continuous shedding at the apical villi is thought to proceed in a caspase-dependent manner (38, 40, 42). Indeed, immunostaining for cleaved caspase-3 in intestinal sections from WT mice revealed that apical cells, about to be released from the top of the villi into the gut lumen, display mostly positive signals (Fig. 2*A*).

Therefore, we aimed to quantify cell loss at the top of the villi by confocal microscopy. To document the shedding events at a sufficiently high number, thick sections of the small intestine (90 µm) were examined at different intestinal regions. By combined staining for actin cytoskeleton and active caspase-3, we visualized the process of cell extrusion with or without apoptosis (Fig. 2*A*). Mechanisms responsible for the removal of cells at the top of the villus either involve apoptosis occurring before shedding through basolateral contraction of actin and myosin during IEC extrusion (40) or live-cell extrusion through mechanical force exerted by neighboring epithelial cells likely followed by anoikis, once the interaction with neighboring cells is lost (38, 39). As expected, the cell loss with an apoptotic phenotype (cleaved caspase-3^+^ cells) was reduced in the intestine of *Casp3/7*^ΔIEC^ mice compared to WT mice (Fig. 2*B*). Some cleaved caspase-3^+^ cells were still detected within the epithelial monolayer of *Casp3/7*^ΔIEC^ mice, which are probably derived from hematopoietic or stromal origin (Fig. 2 *A* and *B*) (38). Alternatively, this could also be due to incomplete Cre-expression across the gastrointestinal tract. Intriguingly, the live-cell extrusion mode of cell shedding was found in both *Casp3/7*^ΔIEC^ and WT mice (Fig. 2*B*). Interestingly, it occurred at a significantly higher rate in *Casp3/7*^ΔIEC^ mice (*P* = 0.0003), compensating the lower level of cleaved caspase-3^+^ shedding (Fig. 2*B*). The total number of shedding cells is similar in both types of mice, indicating that due to the compensatory increase of nonapoptotic cell shedding, the total turnover of IECs is not affected in apoptosis-deficient animals. Compared to WT littermates, ultrastructural analysis of small intestinal apical villi illustrated that most extruding epithelial cells in *Casp3/7*^ΔIEC^ mice displayed a distinct nonapoptotic morphology (Fig. 2*C*). Thus these findings provide genetic evidence for previous morphology-based studies, that IEC shedding occurs both by apoptosis and by an active apoptosis-independent extrusion process via densely packed epithelial cells at the top of the villus (38, 39). Once shed, these cells die equally well, whether they are proficient for executioner caspases or not.

To confirm our TEM findings and start better understanding morphological differences of apical villi, we performed scanning electron microscopy (SEM). WT and *Casp3/7*^ΔIEC^ mice show well-developed extrusion zones on top of the tongue-shaped villus structures. Comparative examination revealed multiple shedding events, vacuolated enterocytes, and incisions between remaining cells in *Casp3/7*^ΔIEC^ mice, while control mice show some shedding cells and intact mucosal surface (Fig. 2*D* and *SI Appendix*, Fig. S4*F*), confirming the difference in the shape of shedding cells. These morphological observations are in line with the hypothesis suggested by the other data of our study, i.e., that alternative nondeath-mediated shedding mechanisms are involved in *Casp3/7*^ΔIEC^ mice.

So far, our results indicated that cell death via the apoptotic executioner caspase-3 and -7 seemed dispensable to guarantee homeostatic turnover in the intestinal epithelium. Still, one possible explanation for the lack of overt phenotypes affecting homeostasis at steady state in *Casp3/7*^ΔIEC^ mice could be the compensatory enhanced activation of alternative caspases or even other cell death modalities such as necroptosis or pyroptosis. We thus tested the possible involvement of such compensatory cell death modalities via Western blotting on cell lysates of WT and *Casp3/7*^ΔIEC^ organoids. WT showed clear activation of caspase-8 and caspase-6 (*SI Appendix*, Fig. S1 *D* and *E*), while this is not the case in *Casp3/7*^ΔIEC^ organoids, arguing that no alternative apoptotic caspases took over the absence of caspase-3 and caspase-7. This conclusion is confirmed by the absence of the 89-kDa PARP1 cleavage, the prototype marker of apoptosis (*SI Appendix*, Fig. S1*G*) (45). Absence of caspase-8 activation sensitizes the necroptotic pathway in the intestine (29, 46). However, neither in WT nor in *Casp3/7*^ΔIEC^ organoids MLKL phosphorylation is observed (*SI Appendix*, Fig. S1*F*), as prototype marker of necroptosis. Moreover, histological analysis of *Mlkl*^ΔIEC^*Casp3/7*^ΔIEC^ triple knockout (TKO) and control mice did not show any signs of dysmorphic architecture as determined by H&E staining and normal lengths of intestinal compartments (*SI Appendix*, Fig. S1 *J*–*L*), supporting the notion that necroptosis apparently is not involved in compensating for the loss of apoptosis in *Casp3/7*^ΔIEC^ mice. Interestingly, GSDME and GSDMD cleavage was not different in cell lysates from WT and *Casp3/7*^ΔIEC^ organoids (*SI Appendix*, Fig. S1 *H* and *I*), suggesting that plasma membrane permeabilizing proteins may still be equally engaged by alternative proteolytic systems in organoids from both genotypes, a subject requiring further investigation.

To examine other possible compensatory signaling pathways in *Casp3/7*^ΔIEC^ mice that could mediate an adaptive alternative developmental reprogramming in the gut in the absence of IEC apoptosis, we performed a transcriptomic analysis of bulk IEC material isolated from both WT and *Casp3/7*^ΔIEC^ mice. Surprisingly, differential gene expression analysis revealed that a substantial number of transcripts were differentially expressed (Fig. 2 *E* and *F*). Assessment of cellular process- and epithelial subtype–specific gene expression signatures via gene set enrichment analysis, revealed that the hallmark “*MYC targets*,” “*Unfolded Protein Response*,” “*E2F Targets*,” “*DNA Repair*,” and “*G2M Checkpoint*” signatures were enriched in *Casp3/7*^ΔIEC^ mice, while WT mice displayed an enrichment of “*IL6-JAK-STAT3*” and “*TGF-beta*” signaling (Fig. 2 *G* and *H*). These results are highly intriguing since they imply the involvement of subphenotypic signaling cues active in *Casp3/7*^ΔIEC^ mice not generating a phenotype in steady state, but maybe forming a basis for a phenotype during challenge. However, these differences did not result in changes in architecture and cellular composition as demonstrated above and as revealed by the absence of any differences based on scRNA-seq comparing the expression of various cell type–specific markers of differentiation (*SI Appendix*, Fig. S4 *B*–*E*). Additionally, we noted that we were unable to uncover these signatures in our scRNA-seq analysis. We thus conclude that in contrast to bulk RNA-seq the transcriptional differences were too nimble to be discovered using scRNA-seq, which harbors an inherent lower coverage of the transcriptome, but that the overall individual cell composition is not changed between IECs from WT and *Casp3/7*^ΔIEC^ mice. Another important aspect is that in contrast to the data obtained from scRNA-seq, cells that were identified to be “dying” or started to display features thereof were excluded from the analysis. Results from the bulk RNA-seq experiment must therefore be interpreted with caution and will be the subject of future lines of investigation examining the role of caspase-3/7 during challenge. Lastly, procedural differences between isolation processes in the scRNA-seq vs. bulk RNA-seq experiments could also play a role in affecting transient ex vivo signatures, otherwise negligible in vivo.

### Loss of Epithelial Apoptosis in the Gut Does Not Affect the Intestinal Microbiome Nor Induce Spontaneous Inflammation during Homeostasis at Steady State.

**Fig. 3.**
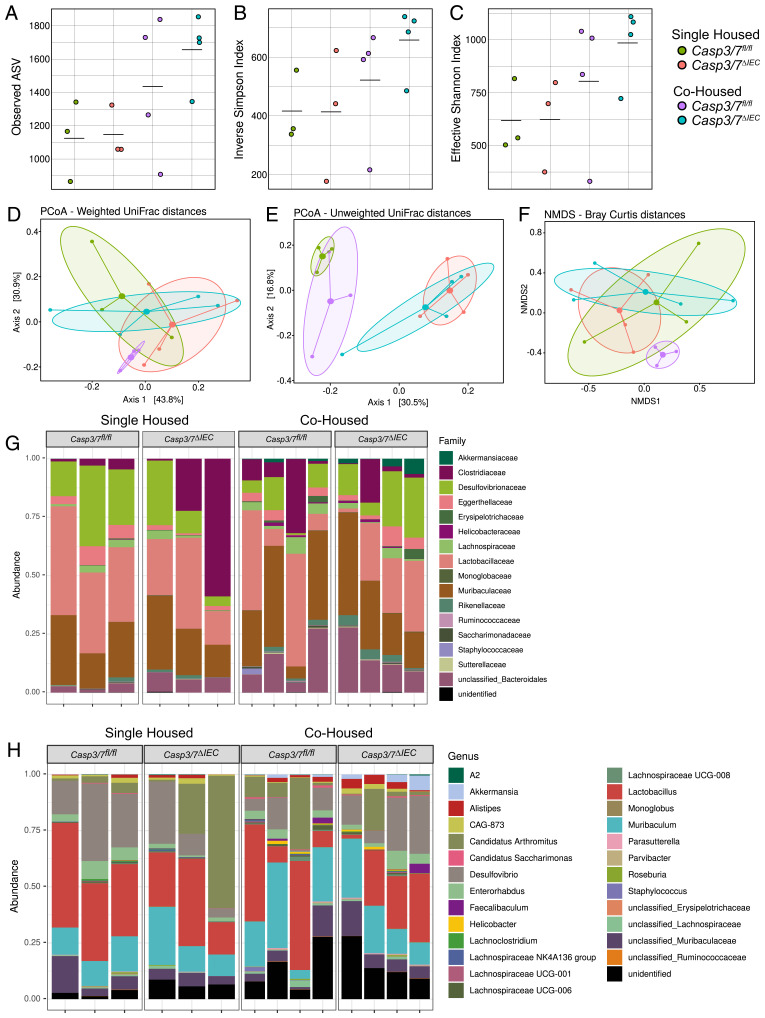
Loss of apoptosis in intestinal epithelial cells does not induce ileal dysbiosis. (*A*) Bacterial richness in the ileum represented by detected (observed) amplicon sequence variants (ASV) of 16S microbiome sequencing. (*B*) Inverse Simpson and (*C*) effective Shannon diversity indices in single- and cohoused *Casp3/7*^ΔIEC^ and *Casp3/7*^fl/fl^ mice revealed no significant differences. (*D*) Principal coordinates analysis of weighted (ADONIS test, R2 = 0.3, adjusted *P* = 1.0); (*E*) unweighted (ADONIS test, R2 = 0.2, adjusted *P* = 1.0) UniFrac distances; and (*F*) nonmetric multidimensional scaling of Bray–Curtis distances (ADONIS test, R2 = 0.6, adjusted *P* = 1.0) show no separation of the *Casp3/7^ΔIEC^* and *Casp3/7*^fl/fl^ mice based on bacterial compositions of ileal samples. Large dots represent group centroids; ellipses indicate the 95% confidence intervals for each group. (*G*) Bar plots of bacterial compositions in ileal samples of single- and cohoused *Casp3/7^ΔIEC^* and *Casp3*/7^fl/fl^ mice at the genus and (*H*) family level.

While we established that the absence of IEC apoptosis exerted no impact on intact intestinal homeostasis at steady state, we sought to assess whether other pillars of intestinal homeostasis such as the microbiome composition and immune system would have been affected. To this end, we first performed 16S sequencing analysis of ileal and colonic microbiome specimens. To dissect genotype-dependent effects on the microbiome, we performed our analysis on samples originating both from mice housed per genotype (these mice were separated according to genotype after weaning) or cohoused with WT littermates. We first assessed bacterial diversity as measured by bacterial richness (observed amplicon sequence variants [ASVs]) and effective Shannon and inverse Simpson indices. None of these metrics reflecting alpha- or beta-diversity was significantly altered in any of the conditions tested (Fig. 3 *A–C* and *SI Appendix*, Fig. S3 *A*–*C*). Supportive of these diversity indices, unsupervised weighted or unweighted principal coordinates, as well as nonmetric multidimensional scaling analysis revealed no difference according to genotype under single- and cohousing conditions (Fig. 3 *D*–*F* and *SI Appendix*, Fig. S3 *D*–*F*). Next, we performed in-depth taxonomic analysis of bacterial communities. Again, no significant changes in microbial architecture were discernible in both ileum and colon (Fig. 3 *G* and *H* and *SI Appendix*, Fig. S3 *G* and *H*). Altogether these findings argued for a negligible role of the executioner caspases-3 and -7 to shape the microbial architecture in the gut during homeostatic conditions independent of housing setup.

**Fig. 4.**
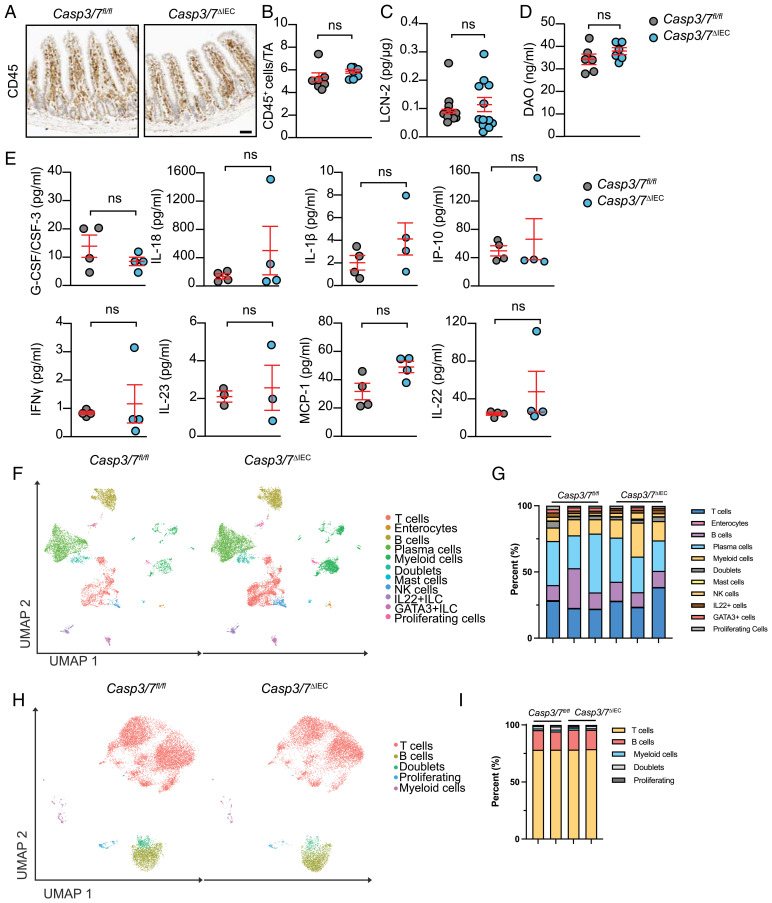
Loss of epithelial apoptosis does not induce spontaneous intestinal inflammation. (*A* and *B*) CD45 staining (leukocyte marker) and quantification in the lamina propria (LP) of small intestine sections from *Casp3/7*^ΔIEC^ (*n* = 7) and *Casp3/7*^fl/fl^ (*n* = 7) mice. (Scale bar, 50 µm.) (*C*) The fecal level of Lipocalin-2 of *Casp3/7*^ΔIEC^ (*n* = 12) and *Casp3/7*^fl/fl^ (*n* = 10) mice. (*D*) The plasma level of DAO in *Casp3/7*^ΔIEC^ (*n* = 6) and *Casp3/7*^fl/fl^ (*n* = 6) mice. (*E*) The plasma level of proinflammatory cytokines in *Casp3/7*^ΔIEC^ (*n* = 3 to 4) and *Casp3/7*^fl/fl^ (*n* = 4) mice. (*F*) UMAP of LP extracted from three *Casp3/7*^fl/fl^ and *Casp3/7*^ΔIEC^ (*n* = 3 per genotype) mice. Identified populations are indicated with relative frequency in *G*. (*H*) Quantification of UMAP of mesenteric lymph nodes (MLNs) extracted from two *Casp3/7*^fl/fl^ and *Casp3/7*^ΔIEC^ (*n* = 2 per genotype) mice. Identified populations are indicated with relative frequency in *I*. (ns: non significant *P*-value.)

To document signs of possible spontaneous inflammation in the intestinal tissue of *Casp3/7*^ΔIEC^ mice, we performed detailed histological analysis and measured markers of inflammation in feces and serum. Histological examination did not reveal elevated infiltration of mononuclear immune cells or other CD45^+^ cells into the mucosal layers of the small intestines of *Casp3/7*^ΔIEC^ mice (Fig. 4 *A* and *B*). Fecal Lipocalin-2 (LCN-2), a marker for intestinal inflammation (47), plasma diamine oxidase (DAO), a marker for intestinal integrity (48), and also plasma levels of proinflammatory cytokines (IL-1β, IL-18, IL-22, IL-23, IFN-γ, G-CSF/CSF3, MIP-1, and IP-10) showed no significant difference between *Casp3/7*^ΔIEC^ and WT mice (Fig. 4 C–E). To systematically document whether the entirety of intestinal immune cell types remained unaffected during epithelia loss of caspase-3/7 we leveraged our scRNA-seq dataset to examine possible differences in deeper mucosal layers comprising largely the lamina propria cells of the small intestine (Fig. 4F) and colon (*SI Appendix*, Fig. S2*G*). In analogy with our previous results on IECs (Fig. 1*N*), UMAP analysis and quantification thereof indicated that the immune cell compartment was largely unaltered in *Casp3/7*^ΔIEC^ mice (Fig. 4 F and G). Additionally, differentially gene expression analysis of individual immune populations comparing WT and *Casp3/7*^ΔIEC^ mice, did not reveal any changes, which would point toward biologically relevant differences in gene signatures in immune cells from small intestine and colon of WT and *Casp3/7*^ΔIEC^ mice. Finally, to document whether intestinal epithelial loss of caspase-3/7 would bear any effects on immune cells draining to lymph nodes (mesenteric), we additionally performed a scRNA-seq analysis of MLN from both WT and *Casp3/7*^ΔIEC^ mice (Fig. 4 H and I). Again, UMAP analysis quantification thereof and differentially expressed genes were unable to document any relevant quantitative or qualitive changes in immune cell reaction and composition between both genotypes.

## Discussion

Apoptosis is the default way of cellular turnover during embryonic development and adult tissue homeostasis. Apoptotic cells release find-me signals such as CX3CL1 that attract macrophages to clear apoptotic cells. This in turn drives two events important for tissue homeostasis: 1) Clearance of apoptotic cells before they undergo secondary necrosis, which may result in inflammatory diseases (5, 49, 50); and 2) it programs phagocytes to produce immunosuppressive mediators such as IL-10 and TGF-beta, which mediate immune suppression and engage other tolerogenic mechanisms like regulatory T cell expansion (17). Therefore, apoptosis is not simply important because of whether it can take place or not, and whether the tissue turnover occurs without it or not, but because its recognition by innate cells such as resident macrophages and microglia cells comprises a crucial mechanism that imparts tissue homeostasis particularly in returning inflammatory or damaged tissue to homeostasis (51, 52). The balance between cell division and apoptosis determines the tissue morphology, architecture, and function (53). Within the intestine, constant turnover of the IECs ensures an intact and effective barrier and tolerance against digested food and the commensal microbiota (54). IECs arise from stem cells at the bottom of the crypts and migrate to the top of the villi in the small intestine. This process takes 4 to 5 d at the end of which IECs are shed into the lumen through mechanisms debated to involve either apoptosis or live extrusion by upwardly moving cells (55). To investigate the role of IEC apoptosis in intestinal physiology in a rodent model, we deleted the executioner caspases-3 and -7 specifically in the intestinal epithelium. Mice were viable and born at expected Mendelian ratios and did not show any sign of burdening over the course of development and into maturity, illustrating that caspase-3 and -7–driven apoptosis is dispensable for intestinal organogenesis and homeostasis at steady state. We performed an in-depth analysis of the entire gastrointestinal tract, including macro and microarchitecture, microbiota, and transcriptomic analyses and did not detect any apparent phenotypic difference between the *Casp3/7^ΔIEC^* transgenic mice and WT littermates (*Casp3/7^fl/fl^*) at the level of development, differentiation, inflammation, and microbiome composition and diversity. The absence of any intestinal or systemic phenotype in *Casp3/7^ΔIEC^* mice indicates that under steady-state conditions, apoptotic cell death is not required for intestinal homeostasis and for keeping a noninflammatory state by continuous sampling of apoptotic IECs by interdigiting phagocytic immune cells, as was elegantly demonstrated by Blander and coworkers (17), but also other mechanisms contribute to immune homeostasis, such as microbiota and their metabolites (butyrate and TLR ligands, for example), and diet (cruciferous vegetables, indoles, and aryl hydrocarbon receptor engagement for example) regulate innate lymphoid cells, dendritic cells (DCs), Treg, and Th17 cells (1, 56–58). Our results with apoptosis-deficient IECs now provide genetic evidence that caspase-3/7–mediated apoptosis apparently is not needed for the continuous turnover of IECs at the top of the villi. We documented the occurrence of a nonapoptotic shedding process in line with the recent findings that extrusion mechanisms at the top of villi precede the appearance of the characteristic readouts of apoptosis such as caspase-3 cleavage and phosphatidylserine exposure (56). Remarkably the process of apoptosis-independent cell extrusion is increased in the absence of caspase-3/7, thereby compensating for the loss of apoptosis and maintaining the total physiological level of cell shedding. Confocal imaging and ultrastructural analysis via TEM and SEM enabled us to detect a concerted shedding release of IECs in what appeared to be a contraction event. It remains to be elucidated which molecular cues precede this alternative mode of shedding. Thus, we conclude that the required balance between apical cell loss at the top of the villus and proliferation in the crypt is retained in *Casp3/7*^ΔIEC^ mice. This demonstrates the resilience, robustness, and adaptivity of the system that apoptosis as a program during tissue development and homeostasis at steady state can be compensated by alternative cell turnover processes. Therefore, analogous to what has been described for the removal of cells within the interdigital webbing during paw development in the absence of Apaf-1 (59), alternative caspase3/7-independent cell loss seems to similarly function as a backup mechanism in the intestine.

The importance of cell death for proper organ development and tissue homeostasis has been emphasized using gene-targeted mice. Notably, seminal work attributed a pivotal role to apoptosis-related molecules such as RIPK1 (60, 61), FADD (62, 63), cFLIP (64, 65), or caspase-8 (29, 65, 66), acting as critical checkpoints in regulation and maintenance of intestinal homeostasis at steady state by preventing aberrant activation of inflammatory cell death by excessive apoptosis or necroptosis. Indeed, conditional knockout of caspase-8 (60) or FADD (63) in intestinal epithelium abrogates intestinal homeostasis by developing epithelial cell necroptosis, loss of Paneth cells, reduced number of goblet cells, and intestinal inflammation, which was prevented by codeletion of essential components of the necrosome such as RIPK3 (63). Similarly, the systemic deletion of caspase-8 in mice led to embryonic lethality, which is prevented by codeletion of essential components of the necrosome pathway, in particular TNFR1, RIPK1, RIPK3, and MLKL (46, 67). These findings established the concept that apoptotic machinery components such as FADD, cFLIP, and caspase-8 are absolutely required for organogenesis by preventing necroptosis (65, 66). This notion erroneously fed the concept that the cell-autonomous process of apoptosis itself controls necroptosis. However, one could better state that the pleiotropic function of the apoptotic initiator caspase-8 and its adaptor FADD and modulator cFLIP control levels of RIPK1 by specific proteolysis of the latter within the molecular complex associated with necrosome, as was recently demonstrated by the inflammatory phenotype of noncleavable RIPK1 (30–32). Our results now refine the insights on how apoptotic pathways control necroptotic pathways. Indeed, the phenotype of *Casp3/7*^ΔIEC^ mice clearly demonstrate that the ablation of the converging point of apoptosis, i.e., the activation of executioner caspase-3/7, apparently does not lead to sensitization to necroptosis during homeostasis and that the necroptosis checkpoint is restricted to caspase-8 proteolytic activity. This is in line with previous reports of sensitization of TNF-induced necroptosis by caspase-8 ablation and not by deficiency or inhibition of other caspases (34).

Apoptosis as a nonimmunogenic programmed cell death modality has been postulated to be essential for intestinal homeostasis by governing the turnover of the intestinal epithelial cells at the top of the villi and therefore shaping the architecture of the gastrointestinal tract (35). Induction of tolerogenic regulatory CD4^+^ T cell differentiation is also attributed to sensing of apoptotic IECs by dendritic cell subsets (65). Therefore, the absence of any spontaneous phenotype in *Casp3/7*^ΔIEC^ mice at the level of inflammation was quite intriguing. Our results suggest that shedding per se and not apoptotic cell death of IECs is sufficient for supporting cellular turnover in the gut.

Given the phenotype of *Casp3/7*^ΔIEC^ mice we wondered whether other genetic ablations reported in the literature would support the same conclusions. In murine studies using *Bok/Bax/Bak* global TKO animals, whose cells are incapable of undergoing intrinsic apoptosis, it was also reported that most animals exhibited severe defects and died shortly after birth, with fewer than 2% TKO mice surviving. Surprisingly, analysis of these mice (died or survived) showed that many developmental processes previously thought to require apoptosis also proceeded normally and that necroptosis, pyroptosis, or autophagic cell death does not substantially substitute for the loss of apoptosis. This observation suggests that morphogenesis can proceed entirely without apoptosis mediated by these proteins (68). Altogether with our findings, this suggests that alternative cell death modalities might take over the function of apoptotic cell death in tissue turnover during development and homeostasis of the intestinal tract at steady state. This illustrates that at least in homeostatic conditions these processes have an intrinsic robustness and redundancy allowing for plasticity and adaptivity of intestinal development, its associated immune system, and the microbiome. However, our data do not exclude a particularly important role of apoptosis in tolerance induction, microbiome shaping, tissue repair by apoptosis-induced proliferation during challenges, and control of intestinal infection. Indeed, a crucial role for apoptosis in IECs was demonstrated during different challenges, e.g., tolerance induction during barrier loss (17), modulation of anticancer immunosurveillance during chemotherapy (59), sensitivity to *Clostridium difficile* infection (58), and IEC apoptosis-dependent boosting of growth of multiple Enterobacteriaceae during dysbiosis (69). Recently the unique role of apoptosis during steady-state regeneration was shown in hair follicle stem cell (HFSC) self-renewal and differentiation. It was shown that deficiency of caspase-9, a crucial apoptotic initiator caspase, leads to accumulation of caspase-3 and inappropriate mitogenic signaling by continuously releasing Wnt3 from apoptotic HFSCs and instructing proliferation (70). Also, in the intestine a link between stem cell proliferation and apoptosis was shown. Following damage in the gut of zebrafish, stem cells (SCs) engulf the apoptotic bodies and activate mitogenic Wnt signaling to maintain homeostatic cell numbers. Inhibition of apoptosis or Wnt signaling abrogated SC proliferation, while overexpression of Wnt8a in combination with apoptosis led to significant increased cell numbers (65). Altogether, a paradigm is emerging that the presence of apoptotic cells during damage and in particular tissues during homeostasis is crucial for SC regeneration, while mice completely lacking crucial regulators (*Bax/Bak/Bok*) can still develop and live normally although at low frequency (68) or mice that lack executioners of apoptosis show normal development, are born at Mendelian frequency, and show at birth a rather limited developmental phenotype (neural tube closure defect, heart anomalies) (33), illustrating that the robustness of the developmental system during organogenesis and development of many tissues is able to cope with deficiency of apoptosis (43).

The fact that apoptosis is dispensable for intestinal development and gut homeostasis under steady-state conditions is supported by the observation that no genetic polymorphisms have been identified implicating caspase-3 or -7 in human intestinal pathology. Alternatively, components preventing the activation of aberrant inflammatory cell death in the gut such as RIPK1 (28), caspase-8 (68), and A20 (71) have been frequently implicated in early-onset intestinal disease. These observations support a conceptual framework in which aberrant inflammatory cell death and subsequent intestinal disorders are kept in check by signaling events distinct from caspase-3 and -7–mediated apoptosis. Taken together our findings demonstrated that under homeostatic conditions, the loss of caspase-3 and -7 within the intestinal epithelium was dispensable for the tissue dynamics of the intestine, the extrusion of IECs at the top of the villi, the microbiome, and the immune cell composition, illustrating the plasticity and robustness of the system to cope with the absence of IEC apoptosis. These data clearly show that caspase-3 and -7–driven apoptosis in the intestinal epithelium is dispensable for gut tissue homeostasis at steady-state conditions, including composition of the intestinal immune system and microbiome in the intestine, illustrating that this apoptotic process can be compensated. This shows that during gut homeostasis at steady state the balance between cell death and cell proliferation can be achieved by redundant processes not driven by caspase-3 and caspase-7 in the IECs. However, the apoptotic modus in IECs might be required during conditions of challenges (17, 58–60), as discussed above.

## Materials and Methods

### Mice.

Caspase-3/7^fl/fl^ and Villin-Cre transgene mice (*Casp3/7^ΔIEC^*) were generated in the VIB-UGent Center for Inflammation Research (IRC) transgenic mice core facility using ES clones HEPDO716_4_G05 and EPD0398_5_E02 (C57BL/6N) from the International Mouse Phenotyping Consortium (IMPC), respectively. The neomycin selection cassette was removed using flippase recombinase codon-optimized deleter mice (72). The breeding was performed with Casp3/7^fl/fl^-Vil1-Cre^tg/wt^ and Casp3/7^fl/fl^-Vil1-Cre^wt/wt^ (73). Mice were bred using heterozygous breeding pairs to obtain wild-type littermate controls. For single-housed experiments, mice were separated according to genotype at weaning. For cohousing experiments mice were housed after weaning irrespective of their genotype. All mice were housed in individually ventilated cages at the VIB Center for Inflammation Research under specific pathogen-free (SPF) conditions. All experiments on mice were conducted according to institutional, national, and European animal regulations. Animal protocols were approved by the ethics committee of Ghent University. Adult mice (8 to 12 wk) from both genders were used for all the experiments.

## Supplementary Material

Supplementary File

## Data Availability

Transcriptomic data have been deposited in the National Center for Biotechnology Information Gene Expression Omnibus (GSE183885).
