# Supplementary material for: Executioner caspases 3 and 7 are dispensable for intestinal epithelium turnover and homeostasis at steady state
Source: Proc Natl Acad Sci U S A. 2022 Feb 1;119(6):e2024508119. doi: 10.1073/pnas.2024508119 (PMC8832966; doi:10.1073/pnas.2024508119)
Supplement: Supplementary File [file pnas.2024508119.sapp.pdf]

Supplementary Information for

Executioner caspases 3 and 7 are dispensable for intestinal epithelium turnover and homeostasis at steady state

Ghazavi F, Huysentruyt J, De Coninck J, Kourula S, Martens S, Hassannia B, Wartewig T, Divert T, Roelandt R, Popper B, Hiergeist A, Tougaard, Vanden Berghe T, Joossens M, Berx G, Takahashi N, Wahida A and Vandenabeele P

Supplementary data: Figure legends: Figure S1 to S5

Supplementary data: Material and Methods (M&M)

M&M S1: Tissue sample preparation, histology, and immunohistochemistry

M&M S2: Generation and culturing of 3D ex vivo cultures

M&M S3: Tissue sample preparation for shedding cell detection

M&M S4: Imaging

M&M S5: Transmission electron microscopy

M&M S6: Scanning electron microscopy

M&M S7: Isolation of cells from mesenteric lymph nodes, small intestine, and colon

M&M S8: Sorting cells for single-cell RNA sequencing

M&M S9: Single-cell RNA sequencing

M&M S10: Single-cell RNA sequencing data analysis

M&M S11: Single-cell barcode demultiplexing

M&M S12: Differential gene expression analysis

M&M S13: DNA extraction from fecal material and 16S sequencing

M&M S14: Epithelial cell isolation, RNA extraction and transcriptomics profiling by bulk RNA sequencing

M&M S15: ProcartaPlex multiplex immunoassay for cytokine and chemokine profiling

M&M S16: Western Blotting

M&M S17: Colonoscopic analysis

M&M S18: Statistics

Supplementary data: Table S1

Supplementary data: References

Supplementary data Figures

Supplementary data: Figure Legends

Supplementary Figure S1. Deletion of Caspase-3 and -7 does not affect intestinal homeostasis at steady state
(A, B) Immunohistochemistry and western blot approaches were used to validate the absence of pro-caspase-3 and -7 in intestinal epithelial cells of $Casp3/7^{\Delta IEC}$ *mice*. (C) Lysozyme (Paneth cell marker) and Ki67 staining (proliferation marker) in the small intestine at three different ages of $Casp3/7^{\Delta IEC}$ and $Casp3/7^{fl/fl}$ littermate mice. (D-I) Western blot analysis for the indicated proteins and their cleavage products from extracts from small intestinal organoids from $Casp3/7^{\Delta IEC}$ with $Casp3/7^{fl/fl}$ mice (n=3 per genotype) (J) representative micrographs from small intestinal sections from $Mlkl^{fl/fl}Casp3/7^{fl/fl}$ and $Mlkl^{\Delta IEC}Casp3/7^{\Delta IEC}$ mice stained via H&E (K & L) quantification of measurement of small intestinal and colonic length per se and relative to body weight (n=6 per genotype).

Supplementary Figure S2. Combined caspase-3 and -7 loss has no impact on colonic homeostasis at steady state
(A) Hematoxylin and eosin staining (H&E) of colon sections from $Casp3/7^{\Delta IEC}$ and $Casp3/7^{fl/fl}$ mice. Scale bars as indicated in the micrographs. (B) Representative image of full-length gastrointestinal tract stretching from the duodenum until the rectum, including jejunum, ileum, stomach, caecum, and colon. (C) and (D) Quantification of Colon length and relative colonic length to body weight of $Casp3/7^{\Delta IEC}$ with $Casp3/7^{fl/fl}$ mice (n=5-6). (E) Representative endoscopic images of the colon from $Casp3/7^{\Delta IEC}$ mice which were aged for 30 weeks. (F) UMAP of colonic intraepithelial cells (IEL) cells extracted from $Casp3/7^{fl/fl}$ and $Casp3/7^{\Delta IEC}$ (n=3 per genotype) mice (G) UMAP of colonic lamina propria cells cells extracted from $Casp3/7^{fl/fl}$ and $Casp3/7^{\Delta IEC}$ (n=3 per genotype) mice.

Supplementary Figure S3. Loss of apoptosis in intestinal epithelial cells does not induce colonic dysbiosis
(A) Bacterial richness in the colon represented by detected amplicon sequence variants of 16S microbiome sequencing, (B) Inverse Simpson and (C) Effective Shannon diversity indices in single- and co-housed $Casp3/7^{\Delta IEC}$ and $Casp3/7^{fl/fl}$ mice revealed no significant differences.
(D) Principal coordinates analysis of weighted (ADONIS test R2= 0.3, adjusted P = 1.0) and (E) unweighted (ADONIS test R2= 0.2, adjusted P = 1.0) UniFrac distances and (F) non-metric multidimensional scaling of Bray-Curtis distances (ADONIS test R2= 0.5, adjusted P = 1.0) show no separation of the $Casp3/7^{\Delta IEC}$ and WT mice based on bacterial compositions of colonic samples. Large dots represent group centroids, ellipses indicate the 95 percent confidence intervals for each group. (G) Bar plots of bacterial compositions in ileal samples of single- and co-housed $Casp3/7^{\Delta IEC}$ and $Casp3/7^{fl/fl}$ mice at the genus and (H) family level.

Supplementary Figure S4. Apical shedding of IECs proceeds independently of Caspase-3 and -7
(A) Heat map showing average expression of different caspases in $Casp3/7^{\Delta IEC}$ (n=4) and $Casp3/7^{fl/fl}$ littermate (n=4) mice. (B-E) Heat maps showing average expression of various epithelial cell markers in $Casp3/7^{\Delta IEC}$ (n=4) and $Casp3/7^{fl/fl}$ mice (n=4) mice (F) SEM micrographs of small intestinal villi from $Casp3/7^{\Delta IEC}$ (n=3) and Casp3/7$^{fl/fl}$ mice (n=3). Magnification as indicated in the figure.

Supplementary Figure S5. Absolute numbers of single cell sequencing
(A) Absolute numbers of immune cell subsets originating from mesenteric lymph nodes. (B) Absolute numbers of immune cell subsets originating from lamina propria of small intestines from $Casp3/7^{fl/fl}$ and $Casp3/7^{\Delta IEC}$ (n=3 per genotype). Cell number is based on the cell types defined in the scRNAseq analysis applied to the total count of viable cells in mLN (n=3 per genotype).

Supplementary data: Material and Methods

M&M S1: Tissue sample preparation, histology, and immunohistochemistry

Freshly isolated small intestines from adult mice were flushed with 4°C PBS to remove the fecal content, subsequently flushed with formalin (4 % formaldehyde in PBS) and fixed overnight in formalin at 4 °C. Next, formalin was removed, and intestines were dehydrated with 70 % ethanol before being processed with the Shandon Citadel tissue processor (Thermo Scientific). The tissue then was embedded in paraffin wax using standard methods. The paraffin-embedded tissue blocks were sectioned at 4 µm thickness with the Micron HM360 Coolcut Paraffin Microtome, air-dried overnight and stained with hematoxylin and eosin using the Thermo Shandon Varistain 24-4 Automatic Slide Stainer (Thermo Scientific). Cover slips were mounted using Entellan mounting medium (Merck Millipore). For Alcian blue staining, 4µm sections were deparaffinized and hydrated using the Shandon Citadel tissue processor (Thermo Scientific). Sections were subsequently incubated in Alcian blue for 30 min and rinsed with water. Cell nuclei were counterstained in 0.1 % nuclear fast red solution for 5 min, washed, dehydrated, and cleared in xylene (in Varistain) before mounting with Entellan mounting medium (VWR International). For immunochemistry, sections were dewaxed and boiled in antigen unmasking solution 100x (Vector, VEC.H-3300) for 20 min in a Pick cell cooking unit, following by a cool down of 2,5 h. Endogenous peroxidase activity was blocked by immersing slides in peroxidase-blocking buffer (3% H_2O_2 in methanol) for 10 min at room temperature. Blocking buffer (5% goat serum and 1% bovine serum albumin in PBS) was added to the slides for 30 min at room temperature. For detection of lysozyme the protocol was slightly different. Before blocking, slides were treated with $NaBH_4$ overnight at 4 °C and then they were blocked overnight with 5 % NGS fish skin gelatin (house-made). Primary antibodies were incubated overnight at 4 °C in blocking buffer (Rabbit anti-CD45, 1/10000, Abcam (ab10558); rabbit anti-Ki67, 1/1000, Cell Signaling (12202S); rabbit anti-procaspase-3, 1/200, Cell Signaling (9662S); rabbit anti-procaspase-7, 1/1000, house-made; rabbit anti-lysozyme, 1/700, Dako (A0099)). Slides were then incubated with a biotinylated secondary antibody (Vector, BA-1000) followed by ABC-method (Vector Laboratories). Detection was done with 3,5-di-amino-benzidine (DAB). Slides were counterstained with Mayer's hematoxylin and mounted in Entellan mounting medium (VWR International). For lysozyme staining, sections were incubated with DyLight-488 conjugated goat anti-rabbit secondary antibody (1:500 dilution, Fisher Bioblock Scientific), and cell nuclei were counterstained with DAPI (40,6-diami- dino-2-phenylindole, Invitrogen) in ProLong Gold anti-fade reagent. Slides were mounted with 1% N-propyl-gallate in glycerol (pH 7) and sealed with nail polish.

M&M S2: Generation and culturing of 3D *ex vivo* cultures

Intestinal *ex vivo* cultures were derived from jejunum/ileum of adult mice as previously described (1). Briefly, a 5 – 10 cm piece of jejunum/ileum of adult mice was dissected and washed in PBS. The intestine was opened longitudinally, villi were scraped away, and the tissue was chopped into 2 – 3 mm pieces. After thorough washing in PBS, the pieces were incubated in 2 mM EDTA/PBS for 30 min at 4 ° C on a rocking platform. The mixture was passed through a 70 µm cell strainer, crypt fractions were isolated and purified by successive centrifugation steps and 50 µl of Matrigel (Corning) was added per 100 – 500 crypts. Drops of crypt-containing Matrigel (25 – 50 µl) were added to pre-warmed wells in a 24-well plate. After polymerization, 250 – 500 µl (10X of Matrigel drop) complete growth medium was added and refreshed every two days. This growth medium contained Advanced DMEM/F12 (Life Technologies, 12634-010), EGF (50 ng/ml, Peprotech), R-Spondin1 (derived from conditioned medium Cultrex® HA-R-Spondin1-Fc 293T, Trevigen) and Noggin (derived from conditioned medium from 293T cells) kindly provided by G. van den Brink (2).

M&M S3: Tissue sample preparation for shedding cell detection

Freshly isolated jejunum/ileum of adult mice were flushed with 4°C PBS to remove the fecal content, subsequently flushed with formalin (4 % formaldehyde in PBS) and fixed overnight in formalin at 4°C. The tissues were washed for 20 min in buffered saline and embedded in low gelling temperature (5 % in PBS) (Sigma, A4018). The agarose-embedded tissue blocks were sectioned at 90 µm thickness with a Leica vibratome in PBS (0.05 % NaN3). The sections were kept at 4 °C in PBS (0.05 % NaN3) before

staining. For the staining, sections were washed 3 times for 5 min in PBT (phosphate buffered saline with 0.5 % bovine serum albumin and 0.1 % triton X-100) and subsequently incubated in blocking buffer for 6h (1 % goat serum in PBT). The blocking buffer was removed, primary antibody (rabbit anti-cleaved caspase-3, 1/50 in PBT, Cell Signaling) and actin co-stain (Acti-stain 488, 1/140 in PBT) were added and incubated overnight at 4 °C. The medium was removed, and the sections were subsequently washed in PBT once 30 min and then 3 times for 10 min. Incubation in secondary antibody (Goat anti-rabbit IgG, Alexa Fluor 594, 1/1000 in PBT) was done overnight at 4 °C in the dark. The sections were washed in PBT (4 x 10 min) and were incubated with Hoechst (1/1000 in PBS) at room temperature in the dark for 30 min. After short wash of section in PBS, they were mounted in 1 % n-propyl gallate in glycerol and were sealed with nail polish.

M&M S4: Imaging

For the shedding experiments, datasets were collected on a Zeiss observer Z.1 microscope equipped with a Yokogawa disk CSU-X1 (Zeiss, Zaventem, Belgium) using a Plan-Apochromat 20X/0.80 NA in combination with a Photometrics Prime 95B camera. Per condition z-stacks of 50-100 µm were imaged with a z-interval of 39 µm. Parameters such as detector gain, laser intensity, exposure time, and image post-processing were kept consistent between the different conditions. Confocal images were represented as maximum projections. Live images from organoids were acquired with an Olympus CKX53 microscope with phase contrast, using a Plan-Apochromat 4X/(Numerical aperture) and 10X/(Numerical aperture). Immunohistochemistry imaging was performed with a Zeiss AxioScan Z. (Carl Zeiss Microscopy, Jena), using a Plan-Apochromat 10X/0.45 NA in combination with either a Hitachi HV-F202SCL or a Hamamatsu Orca Flash for brightfield and fluorescence imaging respectively. Quantification of cells of interest in specific tissue areas were done using QuPath v0.1.2 (3).

M&M S4: Transmission electron microscopy

Samples were fixed with 2.5% glutaraldehyde in 0.1 M sodium cacodylate buffer, pH 7.4 (Electron Microscopy Sciences, USA) for at least 24 h. Thereafter glutaraldehyde was removed, and samples were washed three times with 0.1 M sodium cacodylate buffer, pH 7.4. Post-fixation and prestaining was done for 45 to 60 min with 1% osmium tetroxide (10 ml 4 % osmium tetroxide (Electron Microscopy Sciences, USA)), Samples were washed three times with double distilled water and dehydrated with an ascending ethanol series (15 min with 30%, 50%, 70%, and 90% respectively and two times 10 min with 100%). Subsequently, samples were embedded in Epon (3.61 M glycid ether 100, (Serva Electrophoresis GmbH), 1.83 M methyl nadic anhydride (Serva Electrophoresis GmbH), 0.92 M dodecenylsuccinic anhydride (Serva Electrophoresis GmbH), 5.53 mM 2,4,6-Tris(dimethylaminomethyl) phenol (Serva Electrophoresis GmbH)). 70 nm ultrathin sections were cut at the Reichard-Jung Ultracut E microtome (Darmstadt, Germany) microtome. Ultrathin sections were collected on formvar coated copper grids (Plano, Germany) and automatically stained with Urany-Less EM Stain (Electron Microscopy Sciences) and 3 % lead citrate (Leica, Wetzlar, Germany) using the contrasting system Leica EM AC20 (Leica, Wetzlar, Germany). Imaging was carried out using the JEOL -1200EX II transmission electron microscope (JEOL, Akishima, Tokyo) at 60 kV. Images were taken using a digital camera (KeenViewII; Olympus, Germany) and processed with the iTEM software package (analySIS Five; Olympus, Germany).

M&M S5: Scanning Electron Microscopy

For Scanning Electron Microscopy (SEM) samples were incubated in freshly prepared fixative (2% paraformaldehyde (EMS), 2.5% gluteraldehyde (EMS) in 0.1M Sodium Cacodylate (EMS) buffer, pH7.4) overnight at 4ºC. Fixative was removed by washing 5 x 3 minutes in 0.1M cacodylate buffer and samples were incubated in 2% osmium (OsO_4, EMS) in 0.1M cacodylate buffer for 30 minutes at RT. After washing in H_2O for 3 x 5 minutes, the samples were dehydrated using solutions of increasing EtOH concentration (50%, 70%, 85%, 95%, 2x 100%), for 15 minutes each. Samples were further dehydrated in EtOH:Aceton (1:1) for 15' followed by 100% Aceton for 15'. The samples were then dried in a critical point dryer (Leica EM CPD300) and mounted on an aluminium stub (EMS) using carbon adhesive tape (EMS). Samples were coated with 5nm of Platinum (Quorum Q150T ES). SEM

imaging was performed using a Zeiss Crossbeam 540. Image analysis was carried out in accordance with (4).

M&M S6: Isolation of cells from mesenteric lymph nodes, small intestine, and colon

The mesenteric lymph nodes (mLN) were harvested and stored on ice in PBS containing 3% heat-inactivated FCS. For preparation of single cell suspensions, the lymph nodes were smashed on top of a 70 µm cell strainer using the plunger of a 3-ml syringe. Single cells were collected by centrifugation at 500 g at 4°C and further processed for cell sorting. Small intestines and colons were dissected and flushed with RPMI containing 5% heat-inactivated FCS to remove feces and mucus. Peyer’s patches were removed, and the intestines were cut open longitudinally and subsequently cut transversely into 0.5 cm pieces. For isolation of immune cells within the epithelial layer, the fragments were incubated in HBSS supplemented with 10 mM HEPES, 25 mM NaHCO3, 10% FCS and 1 mM DTE (Sigma-Aldrich, D8255) for 20 min on a shaker at 37°C. This was repeated once, and the resulting cells were collected by centrifugation at 500 g. The cell pellet was resuspended in 37.5% Percoll (Amersham biosciences) and centrifuged at 700 g at RT for 10 min. The top layer was gently removed followed by the remaining Percoll supernatant and the pellet containing intra-epithelial lymphocytes (IEL) and a fraction of epithelial cells was processed further for cell sorting. The remaining tissue pieces of small intestine and colon were further incubated in HBSS containing 5 mM HEPES and 1.3 mM EDTA for 20 min on a shaker at 37°C to remove the remaining epithelial cells, and digested in RPMI supplemented with 10% FCS, 1 mM MgCl2, 1 mM CaCl2, 40 µg/ml DNAse I (Roche, 10104159001) and collagenases depending on the tissue: 100 U/ml collagenase I (Gibco, 17100-017) for small intestine and 0.75 mg/ml collagenase D (Roche, 11088866001), 0.425 mg/ml collagenase V (Sigma-Aldrich, C9263) and 1 mg/ml Dispase (Gibco, 17105-041) for colon. This digestion was performed during 45 min on a 37°C shaking incubator after which the cells were collected by centrifugation at 500 g, resuspended in 37.5% Percoll and centrifuged at 700 g at RT for 10 min. The resulting cell pellet of lamina propria lymphocytes (LPL) was processed further for cell sorting.

M&M S7: Sorting cells for single-cell RNA sequencing

Single cells isolated from mLN, small intestine and colon were stained for CD45 (clone 30-F11) and DAPI during 30 min at 4°C. Sorting of live immune cells (DAPI- CD45+) and live epithelial cells (DAPI- CD45-) was performed using BD FACSAria™ II and BD FACSAria™ III cell sorters.

M&M S8: Single-cell RNA sequencing

Sorted single-cell suspensions were resuspended at an estimated final concentration of 1000 cells/µl and loaded on a Chromium GemCode Single Cell Instrument (10x Genomics) to generate single-cell gel beads-in-emulsion (GEM). Biological replicates were multiplexed per lane using TotalSeq-A Cell Hashing Antibodies. The scRNA libraries were prepared using the GemCode NextGEM Single Cell 3’ Gel Bead and Library kit, version 3.1 (10x Genomics, Cat. PN-1000121) according to the manufacturer’s instructions. The cDNA content of pre-fragmentation and post-sample index PCR samples was analyzed using the 2100 BioAnalyzer (Agilent). Sequencing libraries were loaded on an Illumina NovaSeq flow cell at VIB Nucleomics core with sequencing settings according to the recommendations of 10x Genomics, pooled in a 90:10 ratio for the gene expression and hashtag antibody-derived libraries, respectively.

M&M S9: Single-cell RNA sequencing data analysis

In total we processed eight samples of which the FASTQ files containing raw sequencing reads were processed using the CellRanger v6.0.0 pipeline with default parameters. Reads were aligned to the GRCm38 mouse reference genome. Table S1 shows global CellRanger output metrics, number of hashtags and number of cells per sample. The resulting count matrix was next filtered based on three metrics: number of genes per cell, number of unique molecular identifiers (UMI’s) and percentage of mitochondrial reads. We filtered out low quality cells if the number of genes was <200 or > 8000, if the number of UMI’s was < 100 or > 30000 and finally if cells had more than 20% mitochondrial reads. The filtered data was next processed using Seurat v3 (5) in R version 3.6.0. The data was log-normalized and transformed, top 2000 highly variable genes were detected and cell cycle scores were calculated using the CellCycleScoring function on the Seurat R package (5). Next, the data was scaled and we

regressed out cell cycle effects using ScaleData on the Seurat R package (5) to reduce unwanted variation in the data. Principal component analysis was performed on the highly variable genes to reduce the dimensionality of the data. Finally, unsupervised clustering was performed, and the data was visualised in two-dimensional scatter plots with Uniform Manifold Approximation and Projection (UMAP). Finally, cells were annotated using a combination of well-known markers genes from the literature and differential gene expression analysis per cluster. When obtaining signatures from certain clusters, we also used Enrichr (6-8) to detect certain specific cell type predictions. We subsetted immune cell populations such as lymphoid and myeloid cells and reclustered them to have a more detailed cell annotation for each cell subtype.

We have determined the statistical power of the scRNAseq experiment, by using the SCOPIT tool (https://alexdavisscs.shinyapps.io/scs_power_multinomial/) (9). In the scRNAseq analysis of the small intestine IEL which has the highest number of subpopulations (ten), we needed to sequence 1,461 cells to reach sufficient power to be able to adequately determine the cell composition of these ten observed subpopulations. In the actual scRNAseq experiment, we sequenced in total 30,552 cells (14,461 WT & 16,091 DKO cells) and retained after quality filtering 19,540 cells (7,584 WT & 11,956 DKO cells) (Supplementary data: Table S1), thus sequencing 10x more than needed to reach sufficient power, strengthening our conclusion that there is no difference between the WT and DKO with regard to cell composition of the population studied. Even though the colon IEL has the least number of cells sequenced (with 7 clusters), the number of cells in each cluster is still more then sufficiently high, so we need much less cells to be sequenced in total to reach sufficient power.

M&M S10: Single-cell barcode demultiplexing

TotalSeq-A Cell Hashing Antibody data was normalized using the centered log ratio (CLR) normalization method on the Seurat R package (5). Next, we used the MultiSeqDemux algorithm (10) with auto thresholding between a quantile range of 0.05 - 0.95 to detect negatives, doublets and individual HTO predictions in all cells.

M&M S11: Differential gene expression analysis

Differential gene expression was assessed with the Wilcoxon rank sum test as implemented in the FindMarkers function on the Seurat R package (5). Multiple testing correction was applied using false discovery rate (FDR) which was calculated on the p values from the differentially expressed genes. Genes with FDR < 0.05 were considered to be significant differentially expressed genes.

M&M S12: DNA extraction from fecal material and 16S sequencing

DNA was isolated from 0.25 g frozen ileal and colonic contents using RNeasy PowerMicrobiome kit (Qiagen) according to the manufacturer's instructions, with modifications. The DNase steps (steps 12–16) were not performed and an additional heating step of 95°C for 10 min after step 4 was added to increase the DNA yield. Isolated DNA was subsequently sent to BaseClear B.V. for 16S rRNA gene (V3-V4) PCR amplification using the 341F/785R primers, barcoding, library preparation and 250 bp paired-end Illumina MiSeq sequencing. Analysis of microbiome sequencing data was done in R (4.0.0). Here, exact amplicon sequence variants (ASV) were detected from unprocessed per-sample paired-end fastq files using the DADA2 (2 1.16) package. After quality filtering allowing a maximum of 2 expected errors per read and trimming of forward and reverse reads to 260 and 240 bp respectively, sequence variants were inferred from pooled reads including singletons (39,824 ± 6,272) resulting in a total of 8,826 ASVs. Subsequently, forward, and reverse reads were merged followed by removal of chimeric sequences. Taxonomy was assigned to identified ASVs using the IDTAXA classifier of the DECIPHER (2.16.1) package together with a modified Silva 138 release database. A Phylogenetic tree was generated with FastTree version 2.1.11 after multiple sequence alignment using DECIPHER. The phyloseq package (1.32.0) was used to calculate alpha diversity measures and analyse beta diversity bynon-metric multidimensional scaling of bray-curtis distances and principal coordinates analysis based on weighted and unweighted UniFrac distances. All plots were generated using the ggpubr package (0.4). Permutational Multivariate Analysis of ordination analyses were conducted by applying the ADONIS test using the pairwiseAdonis package (11). Compositional differences between groups were analyzed by Linear Discriminant Analysis (LDA) within the MicrobiotaProcess (1.3.0) package (12) applying a p-value cutoff of 0.05.

We prepared DNA for 16S sequencing from mice in a co-housing experiment in which the two different genotypes (4 vs 4 animals) were housed together irrespective of their genotype ("co-housed"). This experiment did not reveal major differences in richness and composition of microbiome based on diversity or taxonomic analyses. As demonstrated earlier (13), the caging or co-housing effect on the variation of the microbiome in mice is independent of - and thus additive to - the variation due to genetic the background of mice. As such, rather than performing the same "co-housed" experiment we challenged the null hypothesis of no difference between the two genotypes by a different experimental setup in which mice of the two genetic backgrounds were put in separate cages, "single housed" according to genotype, a condition that is enhancing the chance for possible microbiome differences due to different genetic backgrounds. Analysis of the microbiome of this experimental setup, where potential genetic effects would be boosted by the co-housing, again did not reveal any differences according to the genotype of the mice. Overall, PCA analysis of all mice (co-housed or single housed) revealed no differences between the two genotypes on top of the individual variation between mice.

M&M S13: Epithelial cell isolation, RNA extraction and transcriptomics profiling by bulk RNA sequencing

Briefly, a 10 cm piece of jejunum/ileum of adult mice was dissected and washed in Hanks Buffered Saline Solution (HBSS) containing 2% fetal bovine serum (FBS). Peyer patches were removed, the intestine was opened longitudinally, and the tissue was chopped into 2 – 3 mm pieces. Intestinal epithelial cells were isolated by disruption of the structural integrity of the epithelium using EDTA. Jejunum/ileum segments were incubated 2 times in isolation buffer (HBSS without Ca^{2+} and Mg^{2+}, 5 % FCS, 2 mM EDTA) at 37 °C with agitation for 15 minutes. After each incubation, digests were vigorously shaken, and cell suspension filtered by passing the mixture through a 70 µm cell strainer. Purity of individual IEC fractions was analyzed by flow cytometry. PE/Cyanine7 anti-mouse CD326 (Ep-CAM) antibody (1/800, Biolegend), Anti-CD45-AF488 (1/400, Invitrogen) and DAPI (1/800, Invitrogen) were used to sort live $EPCAM^{+}/CD45^{-}$ epithelial cells. The cells were sorted directly in RLT (Qiagen) and RNA was isolated from purified small intestinal IECs using RNeasy micro kit (Qiagen) according to the manufacturer's instructions. Isolated RNA was subsequently used for RNA-seq sequencing and analysis. Libraries were constructed using the Illumina TruSeq RNA Preparation Kit. RNA sequencing was performed at the VIB Nucleomics Core using NextSeq500 sequencer (Illumina) with following parameters: High Output v2.5, 75 cycles (1.1 pM + 1.79 % PhiX v3), Single Reads (76-8-8-0). All samples passed quality control based on the results of FastQC (v0.11.9). Reads were mapped to the mouse reference genome (mm10) via HiSat2 (v2.2.0) and counted via FeatureCounts (v2.0.0). The R package limma (v3.42.2) was used to normalize the data and to perform differential expression analysis. Genes that did not reach a count per million (cpm) value > 1 in at least 4 samples were removed. As such, we ended up with 12309 genes. Differentially expressed (DE) genes were defined based on a log2FC higher than 1 or lower than −1 and adjusted p value (Benjamini–Hochberg (BH) multiple test correction method) lower than 0.05. For the heatmap we first transformed the normalized expression table as 'log2(2^expTable + 1)' and subsequently scaled the values per gene by calculating the mean expression per gene and then subtracting that mean value of each expression value. GSEA 4.1.0 was used to calculate enrichment for the indicated signatures. All signatures were derived from MSigDB.

M&M S14: ProcartaPlex multiplex immunoassay for cytokine and chemokine profiling

Plasma from *Casp3/7*$^{\Delta IEC}$ and WT littermates was obtained by centrifugation of anticoagulated blood at 2,000 g for 10 min at 4 °C. Cytokine and chemokine levels were analyzed using an Affymetrix ProcartaPlex Mouse Magnetic 15-plex (ThermoFisher) according to the manufacturer's instructions on a Bio-Plex 200 device, and data were evaluated using GraphPad Prism 8 software.

M&M S15: Quantification of Fecal LCN-2 and serum DAO by ELISA

Freshly collected fecal samples were reconstituted in PBS and vortexed using Precellys 24 homonizer (Bertin instrument) at 6800 rpm, 2 cycles of 25 sec and 15 sec intervals to get a homogenous fecal suspension. These samples were then centrifuged for 15 min at 11,000 g and 4 °C. Clear supernatant was collected and stored at −20°C until analysis. LCN-2 levels were estimated in the supernatant using DuoSet Mouse Lipocalin-2/NGAL ELISA kit (R&D Systems, DY1857-05) according to

manufacturer's instructions. Plasma was obtained from *Casp3/7*$^{\Delta IEC}$ mice and WT littermate. DAO level was measured using Mouse DAO (Diamine Oxidase) ELISA Kit (BIOMATIK, EKE61613) according to manufacturer's instructions on a Bio-Plex 200 device, and data were evaluated using GraphPad Prism 8 software.

M&M S16: Western Blotting

Intestinal epithelial cells were lysed with 1x Laemmli buffer containing 50 mM Tris-HCl pH 6.8, 2 % SDS and 10 % glycerol. After boiling the samples at 95 °C for 10 min, proteins were separated by SDS–PAGE and transferred to a nitrocellulose membrane (Protran 0,45 micron). Western blotting was performed with the following primary antibodies, overnight at 4 °C (unless otherwise stated): anti-caspase-7 (1/2000, VIB core), anti-caspase-3 (1/2000, VIB core) and HRP-linked anti-beta-tubulin (1/15,000, 1 h room temperature, Abcam ab21058) and secondary antibody, Amersham ECL HRP-linked donkey anti-rabbit IgG (1/3000; NA934 GE Healthcare). Anti-caspase-8 (1/1000, Abnova MAB3429), anti-cleaved caspase-8 (1/1000, Cell Signaling 9429), anti-PARP (1/1000, Cell Signaling 9532S), anti-cleaved PARP (1/1000, Cell Signaling, 9544S), anti-GSDME (1/1000 Abcam, ab215191), anti-GSDMD (1/1000, Abcam ab209845), anti-MLKL (1/1000, Millipore MABC604), anti-phospho S345 (1/1000, Abcam, ab196436) and secondary antibody Amersham ECL HRP-linked goat anti-rat IgG (1/3000, NA935 GE Healthcare). Detection was performed with the western lightning chemiluminescent reagent plus kit (NEL105001EA, PerkinElmer).

M&M S17: Colonoscopic analysis

High-resolution mouse endoscopy was performed as previously described (14) with a 'Coloview' endoscopic system (Karl Storz, Tuttlingen, Germany), consisting of a miniature endoscope, a light source and an air pump to inflate the colon in a regulated manner to facilitate visualization of the bowel. This system allowed investigation of 4 – 5 cm of the distal colon. Mice were anesthetized with 2–2.5% isoflurane in oxygen during endoscopy.

M&M S18: Statistics

Paneth cells, goblet cells, CD45$^+$ cells and Ki-67$^+$ cells were quantified based on the selected region of interest in QuPath software, relative to the chosen area. All data represent at least three biological replicates. Results are expressed as the mean ± SEM. Statistical differences between experimental groups were analyzed using a two-tailed unpaired Student t-test with GraphPad Prism 8 software and were considered significant when $p < 0.05$. Caspase-positive and negative cell shedding were quantified manually and blinded and were analyzed using two-tailed unpaired Student t-test, considered significantly different when $p < 0.05$.

Supplementary data: Table S1

Suppl Table 1: Overview of samples of scRNAseq experiment

sample.id	description	sample	organ	cell origin	state	n.hashtags	n.cells.raw	n.cells filtered	n.read/cell	n.genes/cell
FGH001	Small Intestine IEL Wild type	FGH001	SI	IEL	WT	3	14461	7584	21250	796
FGH002	Small Intestine IEL Caspase 3/7 DKO	FGH002	SI	IEL	DKO	3	16091	11956	23709	1391
FGH003	Small Intestine LPL Wild type	FGH003	SI	LPL	WT	3	8236	6907	21452	1210
FGH004	Small Intestine LPL Caspase 3/7 DKO	FGH004	SI	LPL	DKO	3	13822	11983	28322	1548
FGH005	Colon IEL+LPL Wild type	FGH005	Colon	IEL+LPL	WT	6	9786	6997	24362	1313
FGH006	Colon IEL+LPL Caspase 3/7 DKO	FGH006	Colon	IEL+LPL	DKO	6	7189	5445	19218	1243
FGH007	Mesenteric Lymph Nodes Wild type	FGH007	LN	mLN	WT	3	14488	13797	8528	914
FGH008	Mesenteric Lymph Nodes Caspase 3/7 DKO	FGH008	LN	mLN	DKO	3	11365	10987	5041	640

SI = small intestine; IEL = intestinal epithelium leukocytes; LPL = lamina propria leukocytes; mLN = mesenteric lymph node leukocytes

Supplementary data: References

1. Sato, Toshiro, Robert G. Vries, Hugo J. Snippert, Marc van de Wetering, Nick Barker, Daniel E. Stange, Johan H. van Es, et al. 2009. "Single Lgr5 Stem Cells Build Crypt-Villus Structures in Vitro without a Mesenchymal Niche." *Nature* 459 (7244): 262–65.
2. Heijmans, Jarom, Jooske F. van Lidth de Jeude, Bon-Kyoung Koo, Sanne L. Rosekrans, Mattheus C. B. Wielenga, Marc van de Wetering, Marc Ferrante, et al. 2013. "ER Stress Causes Rapid Loss of Intestinal Epithelial Stemness through Activation of the Unfolded Protein Response." *Cell Reports* 3 (4): 1128–39.
3. Bankhead, Peter, Maurice B. Loughrey, José A. Fernández, Yvonne Dombrowski, Darragh G. McArt, Philip D. Dunne, Stephen McQuaid, et al. 2017. "QuPath: Open Source Software for Digital Pathology Image Analysis." *Scientific Reports* 7 (1). https://doi.org/10.1038/s41598-017-17204-5.
4. Skrzypek, T. H., W. Kazimierczak, H. Skrzypek, J. L. Valverde Piedra, M. M. Godlewski, and R. Zabielski. 2018. "Mechanisms Involved in the Development of the Small Intestine Mucosal Layer in Postnatal Piglets." *Journal of Physiology and Pharmacology: An Official Journal of the Polish Physiological Society* 69 (1): 127–38.
5. Stuart, Tim, Andrew Butler, Paul Hoffman, Christoph Hafemeister, Efthymia Papalexi, William M. Mauck 3rd, Yuhan Hao, Marlon Stoeckius, Peter Smibert, and Rahul Satija. 2019. "Comprehensive Integration of Single-Cell Data." *Cell* 177 (7): 1888-1902.e21.
6. Chen, Edward Y., Christopher M. Tan, Yan Kou, Qiaonan Duan, Zichen Wang, Gabriela Vaz Meirelles, Neil R. Clark, and Avi Ma'ayan. 2013. "Enrichr: Interactive and Collaborative HTML5 Gene List Enrichment Analysis Tool." *BMC Bioinformatics* 14 (April): 128.
7. Kuleshov, Maxim V., Matthew R. Jones, Andrew D. Rouillard, Nicolas F. Fernandez, Qiaonan Duan, Zichen Wang, Simon Koplev, et al. 2016. "Enrichr: A Comprehensive Gene Set Enrichment Analysis Web Server 2016 Update." *Nucleic Acids Research* 44 (W1): W90-7.
8. Xie, Zhuorui, Allison Bailey, Maxim V. Kuleshov, Daniel J. B. Clarke, John E. Evangelista, Sherry L. Jenkins, Alexander Lachmann, et al. 2021. "Gene Set Knowledge Discovery with Enrichr." *Current Protocols* 1 (3): e90.
9. Davis, A., Gao, R. and Navin, N.E. 2019. "SCOPIT: sample size calculations for single-cell sequencing experiments." *BMC Bioinformatics 20, 566.*
10. McGinnis, Christopher S., David M. Patterson, Juliane Winkler, Daniel N. Conrad, Marco Y. Hein, Vasudha Srivastava, Jennifer L. Hu, et al. 2019. "MULTI-Seq: Sample Multiplexing for Single-Cell RNA Sequencing Using Lipid-Tagged Indices." *Nature Methods* 16 (7): 619–26.
11. Martinez Arbizu, P. 2020. "PairwiseAdonis: Pairwise Multilevel Comparison Using Adonis." *R Package Version* 0. 4.
12. *MicrobiotaProcess*. n.d. Github. Accessed August 13, 2021. https://github.com/YuLab-SMU/MicrobiotaProcess.
13. Hildebrand, F., Nguyen, T.L.A., Brinkman, B. *et al.* Inflammation-associated enterotypes, host genotype, cage and inter-individual effects drive gut microbiota variation in common laboratory mice. *Genome Biol* **14,** R4 (2013).
14. Becker, C., M. C. Fantini, and M. F. Neurath. 2006. "High Resolution Colonoscopy in Live Mice." *Nature Protocols* 1 (6): 2900–2904.

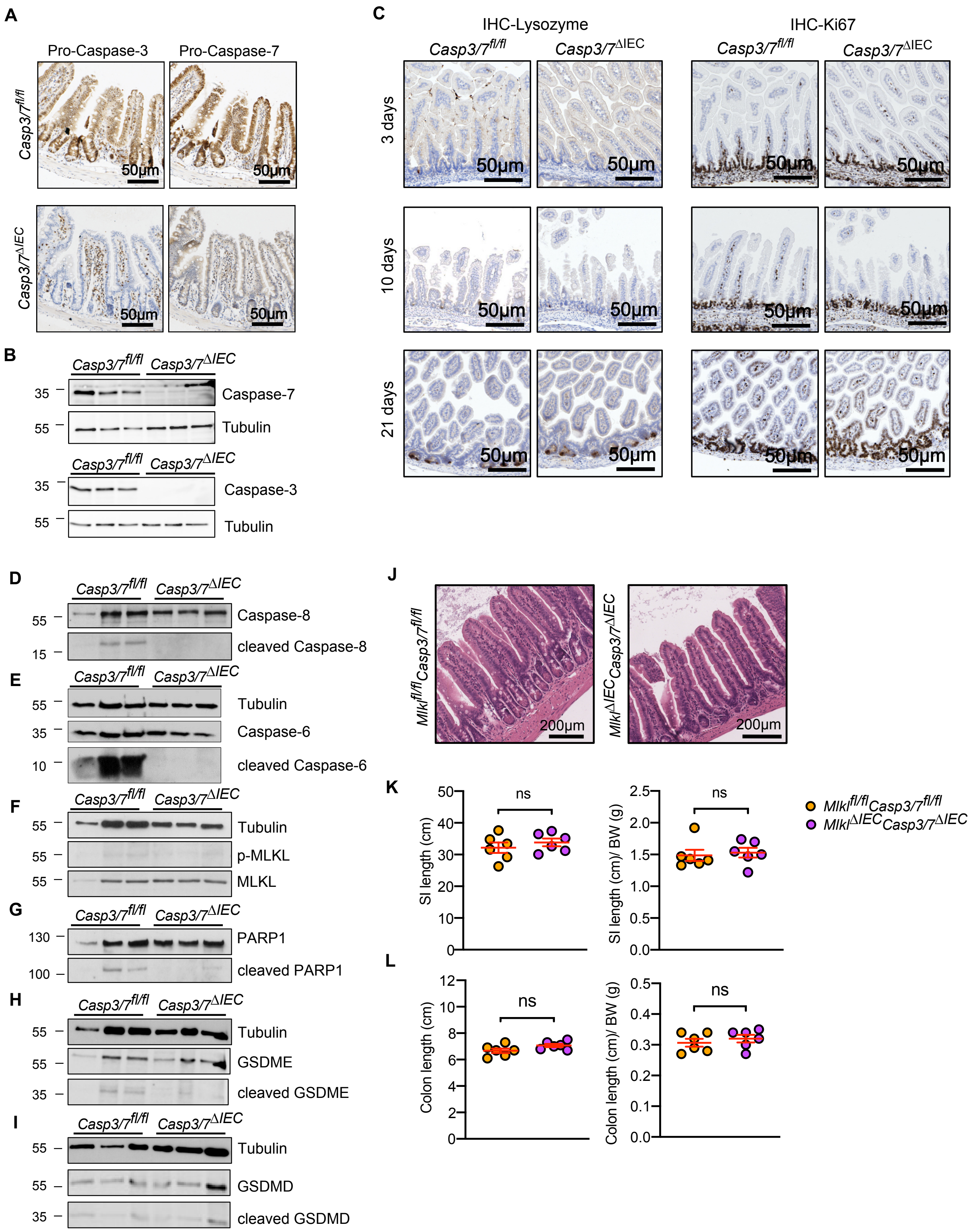

Supplementary Figure 1

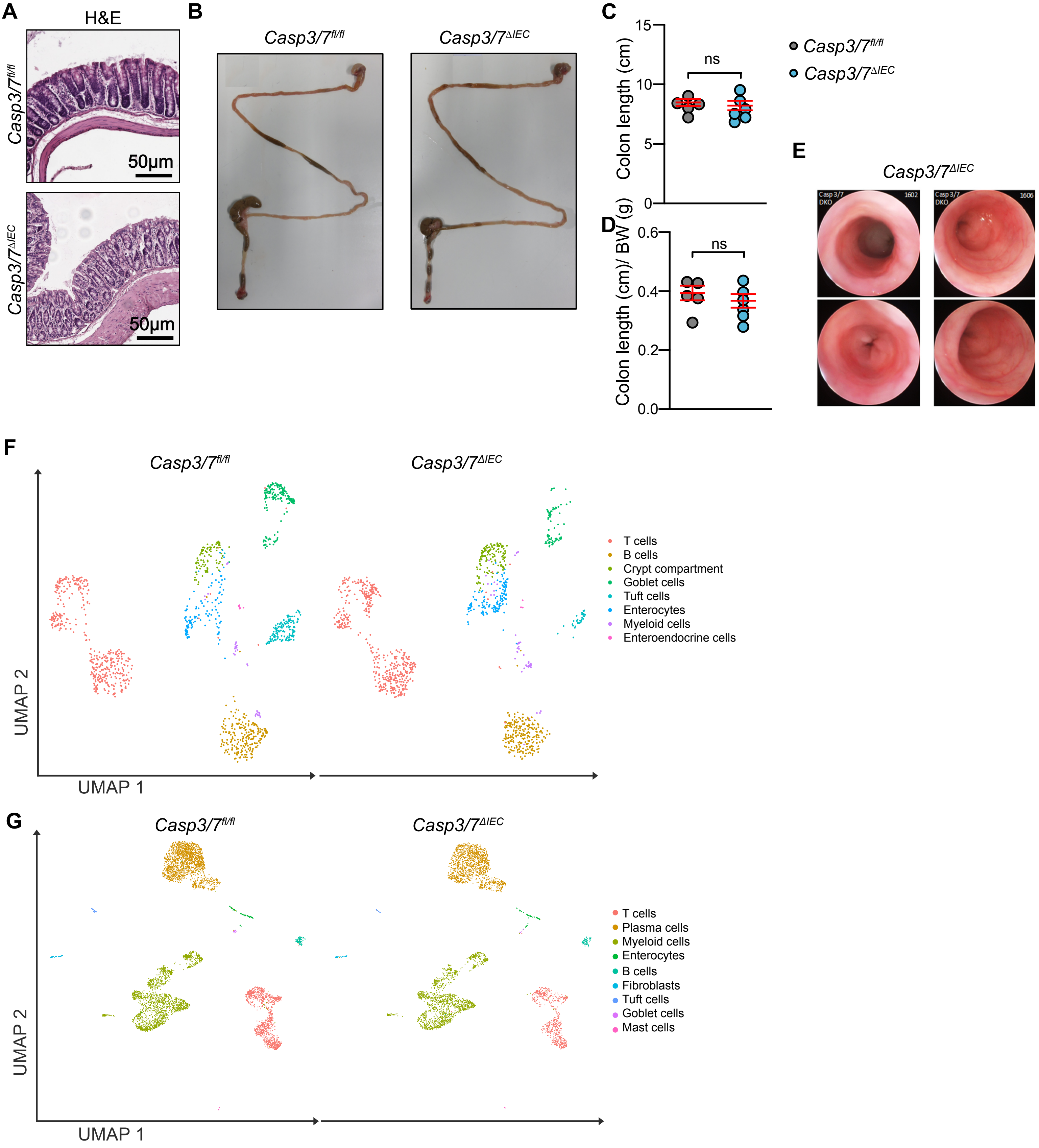

Supplementary Figure 2

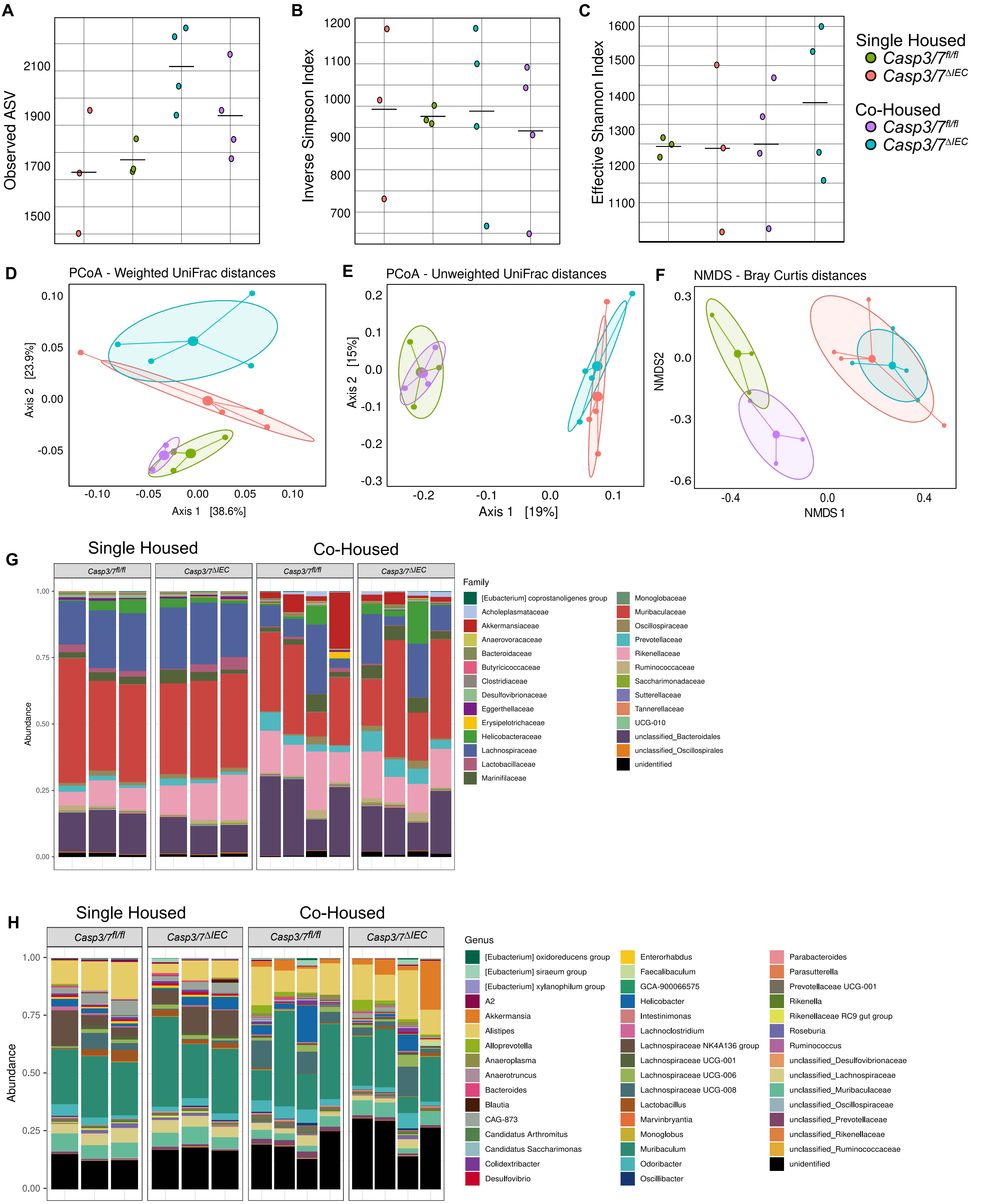

Supplementary Figure 3

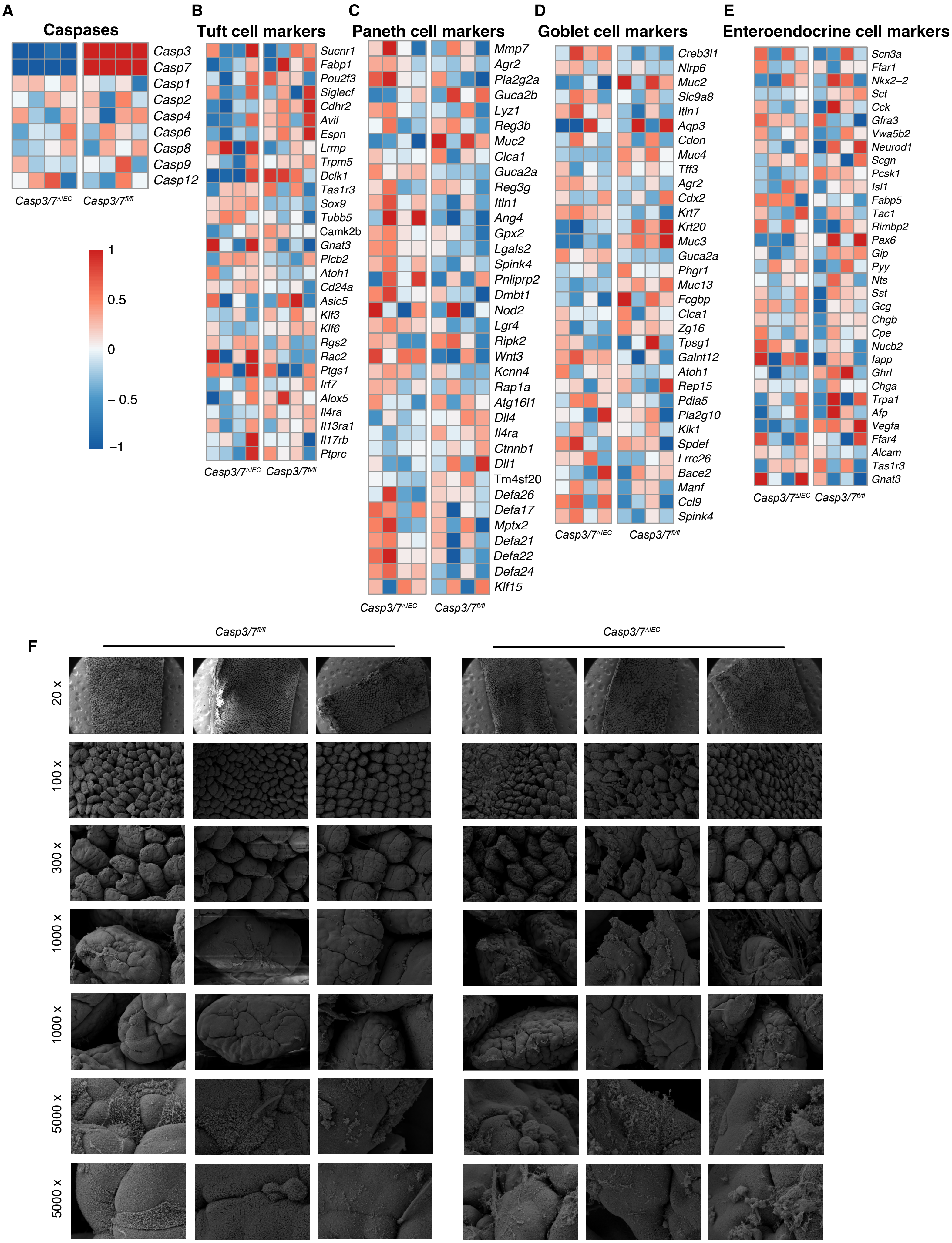

Supplementary Figure 4

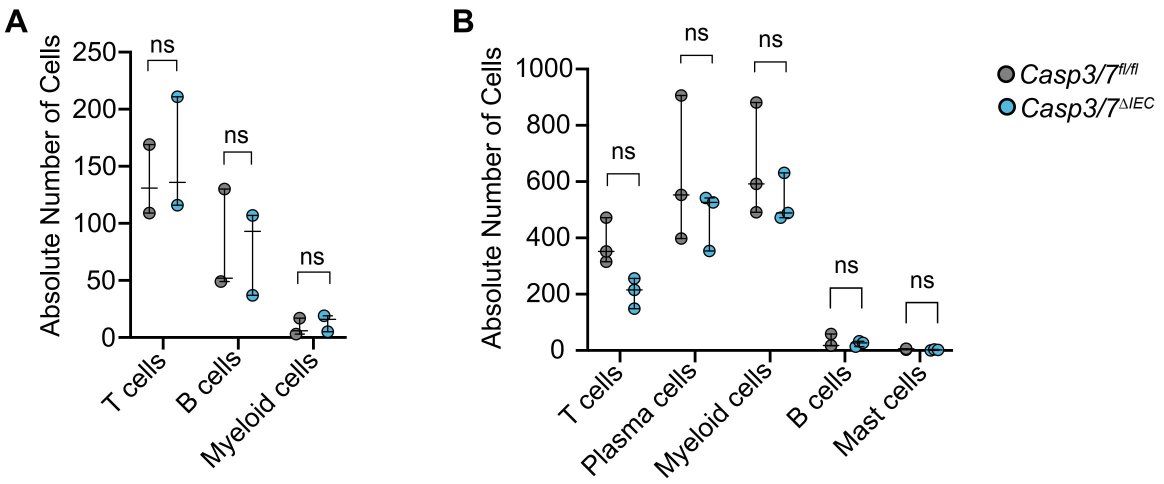

Supplementary Figure 5
